# Chimeric antigen receptor with novel intracellular modules improves antitumor performance of T cells

**DOI:** 10.1038/s41392-024-02096-5

**Published:** 2025-01-15

**Authors:** Pengju Wang, Yiyi Wang, Xiaojuan Zhao, Rui Zheng, Yiting Zhang, Ruotong Meng, Hao Dong, Sixin Liang, Xinyi He, Yang Song, Haichuan Su, Bo Yan, An-Gang Yang, Lintao Jia

**Affiliations:** 1 State Key Laboratory of Holistic Integrative Management of Gastrointestinal Cancers, Department of Biochemistry and Molecular Biology, Fourth Military Medical University, Xi’an, Shaanxi China; 2 Henan Key Laboratory of Immunology and Targeted Therapy, School of Medical Technology, Xinxiang Medical University, Xinxiang, Henan China; 3 Department of Immunology, Fourth Military Medical University, Xi’an, Shaanxi China; 4 Department of Oncology, Tangdu Hospital, Fourth Military Medical University, Xi’an, Shaanxi China

**Keywords:** Drug development, Tumour immunology, Translational research

## Abstract

The excessive cytokine release and limited persistence represent major challenges for chimeric antigen receptor T (CAR-T) cell therapy in diverse tumors. Conventional CARs employ an intracellular domain (ICD) from the ζ subunit of CD3 as a signaling module, and it is largely unknown how alternative CD3 chains potentially contribute to CAR design. Here, we obtained a series of CAR-T cells against HER2 and mesothelin using a domain comprising a single immunoreceptor tyrosine-based activation motif from different CD3 subunits as the ICD of CARs. While these reconstituted CARs conferred sufficient antigen-specific cytolytic activity on equipped T cells, they elicited low tonic signal, ameliorated the exhaustion and promoted memory differentiation of these cells. Intriguingly, the CD3ε-derived ICD outperformed others in generation of CAR-T cells that produced minimized cytokines. Mechanistically, CD3ε-based CARs displayed a restrained cytomembrane expression on engineered T cells, which was ascribed to endoplasmic reticulum retention mediated by the carboxyl terminal basic residues. The present study demonstrated the applicability of CAR reconstitution using signaling modules from different CD3 subunits, and depicted a novel pattern of CAR expression that reduces cytokine release, thus paving a way for preparation of CAR-T cells displaying improved safety and persistence against diverse tumor antigens.

## Introduction

In recent years, chimeric antigen receptor T cells (CAR-T cells) have gained accumulating success in the development of innovative treatment for various cancers.^1^ A typical second-generation CAR consists of a single-chain variable fragment (scFv) of a monoclonal antibody that recognizes tumor antigen, a hinge and transmembrane region from CD8α, a costimulatory domain from CD28 or 4-1BB, and a CD3ζ-derived intracellular domain (ICD).^2,3^ Although CAR-T cells targeting hematologic malignancies have demonstrated potent efficacy and yielded remarkable clinical outcomes in varied types of leukemia and lymphoma, their application in most solid tumors has so far achieved limited benefits, with barriers lying mainly in unsatisfactory persistence and low therapeutic tolerability due largely to excessive inflammatory cytokine release by activated CAR-T cells.^4–6^

Most CAR-T cells fail to display a long-term tumoricidal reactivity because they almost inevitably undergo exhaustion or apoptosis after activation.^7^ Meanwhile, the exhaustion of CAR-T cells can also be elicited by tonic signaling, i.e. constitutive signaling that causes undesirable CAR activation without stimulation by a tumor antigen.^8^ Cytokine release syndrome (CRS) represents another important challenge that hinders its applicability in various malignancies.^6^ Although the interleukin 6 antibody, tocilizumab, frequently accompanied by corticosteroids, has been documented as an effective means to manage CRS, the cytokine storm is hard to be resolved and can be life-threatening when manifesting with other features of systemic inflammation including hypoxia, hypotension and capillary leak.^9^ Given an exceptional complexity of the pathophysiology of CAR-T cell-induced CRS, a multi-step process involving intricate cellular interactions, it is probably more likely to avoid the occurrence of severe CRS through structural optimization of a functional CAR.^9,10^

Despite the diversity of tumor antigens in terms of their density and affinity to the scFv, the intracellular moieties of a CAR convert antigen stimuli to common signals that govern T cell activation and cytokine production.^2,4^ However, while CARs containing a costimulatory domain of CD28 are reported to generally lead to a faster onset of CRS than those composed of a 4-1BB domain, the cytokine-producing abilities of T cells expressing either type of CAR are highly similar.^9^ In the carboxyl terminus of the conventional CAR, the endodomain from ζ chain but not the entire CD3 signalosome is incorporated based on the understanding that the cytoplasmic modules are redundant for transmitting the extracellular signal of antigen engagement.^11,12^ CD3 is a multimeric protein that aid in TCR assembly, trafficking to the cytomembrane, and signal transduction in T lymphocytes.^13^ The CD3 subunits share an extracellular immunoglobulin domain, a type I transmembrane structure and several intracellular segments including ITAMs, although they vary in many characters, such as the existence of other cytoplasmic modules and their detailed functions in mediating cell signaling during T cell maturation and activation.^13,14^ In contrast to other subunits of CD3, CD3ζ in its intracellular region possesses 3 immunoreceptor tyrosine-based activation motifs (ITAMs) each with 2 tyrosine residues in tandem, which exhibit ordered or overlapping phosphorylation after exposure to MHC-presented peptides with varied affinities.^15^ It thus merits investigation whether the ICD encompassing a single ITAM from an individual CD3 chain is sufficient for CAR generation and cytotoxicity of the derived CAR-T cells.

The appropriate architecture of CARs has recently been studied with the attempt to enhance the anti-tumor performance while minimizing the adverse effect of CARs using alternative or modified ICDs.^16–18^ For example, a most recent study has revealed that the endodomains from alternative CD3 subunits can be harnessed for design of effective CARs.^19^ In addition, inclusion of a JAK-STAT signaling domain in CAR intracellular region is effective to facilitate the activation and prevent the terminal differentiation of modified T cells.^17^ A high through-put screening for functional signaling domain configurations has revealed the potential of different activating or co-stimulatory domains in optimizing the ICDs of CARs.^16^ However, the properties of CAR variants with alternative ICDs including those from different CD3 subunits, e.g. T cell activation/exhaustion and inflammatory cytokine production, still lack an in-depth study, and the mechanisms underlying the anti-tumor capabilities of these CAR-T cells are to be unraveled.

In the present study, we generated a series of CARs against human epidermal growth factor receptor 2 (HER2) or mesothelin.^16,20,21^ Unlike classical CARs consisting of the CD3ζ intracellular domain, these CARs were constructed using an intact or modified endodomain from any subunit of the CD3 complex. The tumoricidal activities of resultant CAR-T cells were evaluated, which indicated that these one ITAM-containing CARs were effective and superior to classical CARs in that they elicited a lower tonic signal. Thus, these CAR-T cells displayed mitigated exhaustion and underwent improved differentiation into memory populations. Surprisingly, the CD3ε-derived CAR outperformed other CARs in term of undesirable cytokine production. In a mechanistic study, we observed that this novel class of CARs exhibited a unique self-restrained cytomembrane expression on CAR-T cells, which was orchestrated by a composition of charged amino acids in the ICD.

## Results

### CARs comprising a single CD3 ITAM confer potent cytotoxicity on T cells

**Fig. 1:**
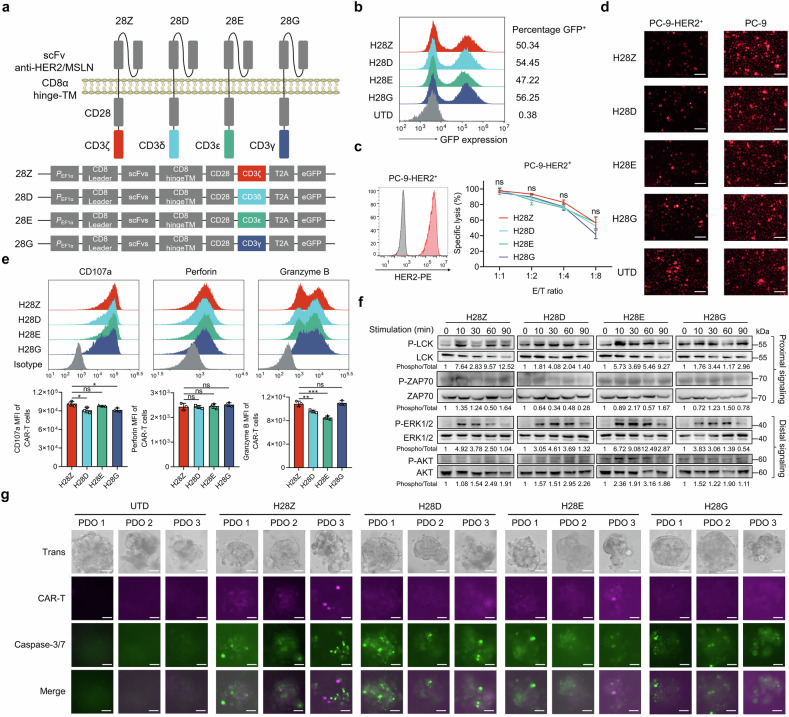
Cytolytic activities of T cells expressing CARs with varied CD3-derived endodomains. **a** Schematic diagram of CARs constructed using varied scFvs and endodomains. **b** Human T cells isolated from PBMCs were infected with recombinant lentiviruses for indicated CARs, and the percentages of CAR-expressing cells were measured through FCM assay for the cotranslated GFP. H28Z refers to T cells expressing a CAR composed of a HER2 scFv, a CD28-derived costimulatory domain and an ICD from CD3 ζ chain, whereas others represent CARs containing an ICD from the δ (D), ε (E) or γ (G) chain of CD3, respectively. UTD, untransduced cells. **c** CAR-T cells were cocultured with PC-9 cells modified to express HER2 at varied E/T ratios for 16 h, and the percentages of cell lysis were calculated and plotted. **d** CAR-T cells were co-cultured with Dil-labeled PC-9 cells or those modified to express HER2 (E:T = 2:1) for 6 h, and cells were observed via fluorescence microscopy. Bar, 100 μm. **e** CAR-T cells and malignant cells were cocultured for 6 h (E:T = 1:1). FCM assay was used to evaluate cell degranulation marked by membrane CD107a levels and to measure the release of cytotoxic granules containing perforin and granzyme B. **f** HER2-targeted CAR-T cells were stimulated by incubation with PC-9 cells overexpressing HER2 for indicated times. Cells were then harvested for Western blot analysis. The intensities of the protein bands were quantified by the ImageJ software, and the ratios of phosphorylated to total protein levels were plotted. **g** Patient-derived organoids (PDOs) of HER2-positive ovarian cancer were established, followed by coculture with 3 × 10^5^ CAR-T cells. The infiltration of CAR-T cells was imaged (red) 48 h later, and neoplastic cells undergoing apoptosis were visualized using a dye-conjugated substrate for active cleaved caspase 3/7 (green). Scale bars, 20 μm. Data are representative images and expressed as the means ± SD of three independent experiments. *P < 0.05, **P < 0.01, ***P < 0.001; ns, non-significant

The scFvs against different tumor-associated antigens including HER2 (H) and mesothelin (M) were used to construct a series of second-generation CARs. These CARs consisted of a costimulatory domain and an intracellular moiety from the CD3 multisubunit complex, e.g. H28Z refers to the CAR composed of a HER2 scFv, a CD28 costimulatory domain and an ICD from CD3ζ (Fig. 1a; Supplementary Fig. 1a). In addition to the conventional CARs with an intact CD3ζ ICD, we also obtained CARs with reconstituted ICDs (ri-CARs) including those with an endodomain that contains merely one of the three ITAM of CD3ζ, e.g. the first (membrane-proximal) ITAM in H28Z1, and also CARs with an ICD of the δ (D), ε (E) or γ (G) chain of CD3. Recombinant lentiviruses co-translationally expressing these CARs and GFP were generated and used to infect T cells prepared from human peripheral blood mononuclear cells (PBMCs), which resulted in efficient expression of CARs on T cells (Fig. 1b; Supplementary Fig. 1b). The cytotoxic activities of CAR-T cells were investigated via co-culture with cancer cells. We observed that all groups of CAR-T cells except H28Z2 were capable of potent lysis of lung carcinoma PC-9 cells transfected to express HER2 (Fig. 1c, d; Supplementary Fig. 1c). Consistently, we observed similar cytotoxicity of T cells expressing a mesothelin-targeted CAR composed of varied CD3 ICDs when cocultured with human cervical cancer HeLa cells that endogenously express mesothelin (Supplementary Fig. 1d, e). The comparable cytotoxicity of these CAR-T cells was also manifested by similar granzyme B and perforin release, and very close levels of surface CD107a, a lymphocyte degranulation marker (Fig. 1e). When cocultured with malignant cells, HER2-targeted CAR-T cells, despite the discriminated ICD configurations of CARs, displayed similar levels of antigen-triggered Lck and ZAP70 phosphorylation, which represents the proximal signaling of T cell activation (Fig. 1f).^22^ Concomitantly, these cells showed almost equivalent capacities of activating canonical downstream pathways required for complete priming and cytotoxicity (Fig. 1f).^22^ Using organoids derived from clinical HER2-positive ovarian cancer specimens, we observed almost indiscriminate rapid infiltration and apoptosis-inducing activities of T cells expressing the conventional or reconstituted CARs (Fig. 1g). Thus, the CD3 endodomain containing a single ITAM can be utilized as a signal module for CARs targeting solid tumors.

### CARs with a single ITAM generate low tonic signal and relieve CAR-T cell exhaustion

**Fig. 2:**
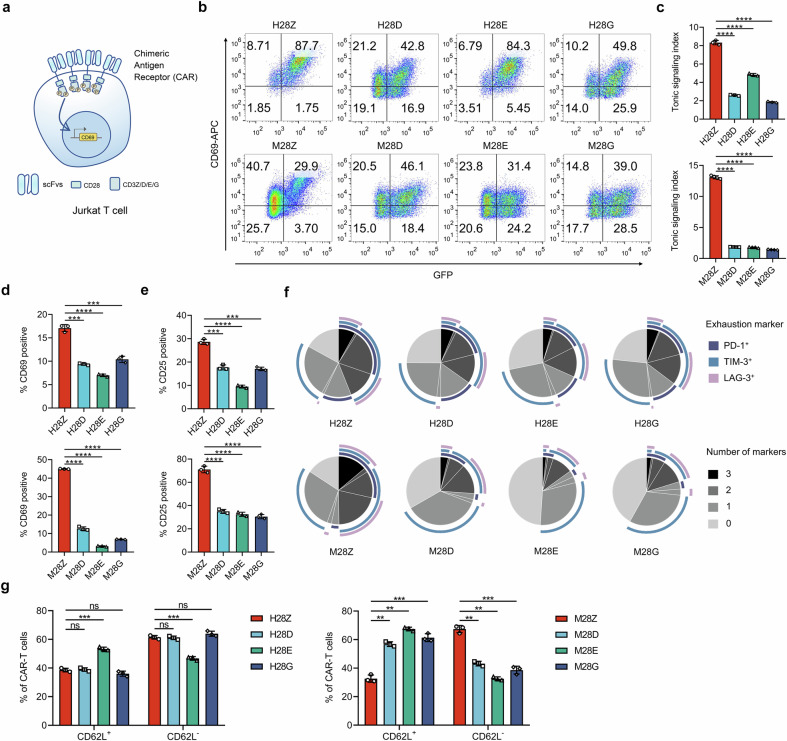
CARs with reconstituted ICDs elicit modest tonic signal and contribute to mitigated CAR-T cell exhaustion. **a** Schematic representation for assay of tonic signals generated by CARs with different configurations. CARs with varied antigen recognition and intracellular domains were introduced into Jurkat cells, and the tonic signal intensities were evaluated through FCM assay of CD69 expression. **b**, **c** CD69 levels on Jurkat cells transduced to express the indicated CARs were measured via FCM, and the percentages of CD69-positive cells were plotted (**b**). The tonic signal index was calculated as the ratios of CD69 MFI to that of GFP, which was co-translationally expressed from the CAR construct (**c**). **d**–**g** Human primary T cells engineered with indicated CARs were subjected to long-term culture (14 d), followed by FCM assay for CD69 (**d**) and CD25 (**e**) expression or for composition of cell populations expressing the indicated surface proteins (**f**, **g**). Data are representative images and expressed as the means ± SD of three independent experiments. **P < 0.01, ***P < 0.001, ****P < 0.0001; ns, non-significant

Tonic signal triggered by CARs in the absence of specific antigens impedes the tumoricidal persistence of CAR-T cells.^23^ We first monitored the tonic/auto-activation of a CAR-modulated human T cell lymphoma cell line, Jurkat, via flow-cytometric detection of CD69 expression (Fig. 2a).^24^ The levels of CD69 in cells transduced to express ri-CARs were remarkably lower than cells expressing the conventional CAR, which was independent of tumor antigens recognized by CARs (Fig. 2b; Supplementary Fig. 2a, b). Consistently, the tonic signal index scored by a mean fluorescence intensity (MFI) ratio of CD69 to GFP was dramatically lower in cells expressing ri-CARs (Fig. 2c; Supplementary Fig. 2c). Then, we measured antigen-independent activation of CAR-T cells after long-term culture and incubation with CD3/CD28 antibody. As a result, when compared to the conventional CAR-T cells, those engineered with ri-CARs displayed substantially reduced expression of CD69 and CD25, both are biomarkers for activated T cells (Fig. 2d, e).^25^ Exhaustion of T cells induced predominantly by tonic signaling represents a pivotal challenge for CAR-T cell therapy.^26^ We found that the immunosuppressive receptors PD-1, Tim-3 and LAG-3, which are indicative of T cell exhaustion,^26,27^ displayed a concomitant lower expression on ri-CAR-engineered T cells than their conventional counterparts (Fig. 2f). Furthermore, these groups of CAR-T cells comprised a larger subpopulation of cells expressing CD62L, which is a marker of naive and central memory T cells (Fig. 2g).^28^ To exploit the mechanisms underlying the different phenotypes of CAR-T cells, we stimulated these cells and performed RNA-seq to dissect mechanisms underlying their discrepant responses to antigen crosslinking. We detected remarkably different gene expression landscapes between CAR-T cells designed using ICDs from different CD3 subunits (Supplementary Fig. 3a, b). KEGG pathway analysis revealed a comprehensive participation of differentially expressed genes (DEGs) in T cell proliferation, TCR signaling and cytokine-related pathways (Supplementary Fig. 3c). Gene set enrichment analysis (GSEA) indicated decreased expression of naive/memory-related genes and elevation in T cell activation- and effector-associated genes in H28Z compared with other groups of CAR-T cells (Supplementary Fig. 3d). Meanwhile, genes regulating T cell effector function, exhaustion and memory differentiation were differentially expressed between H28Z and other CAR-T cells (Supplementary Fig. 3e). Furthermore, we detected a significantly different profile of cytokines expressed by these cells (Supplementary Fig. 3f). Thus, reconstitution of CAR with a single ITAM-containing CD3 endodomain is beneficial to minimizing tonic signaling and exhaustion of CAR-T cells that might eventually affect cytokine production by these primed CAR-T cells.

### Substitution for conventional ICD reduces inflammatory cytokine release by CAR-T cells

**Fig. 3:**
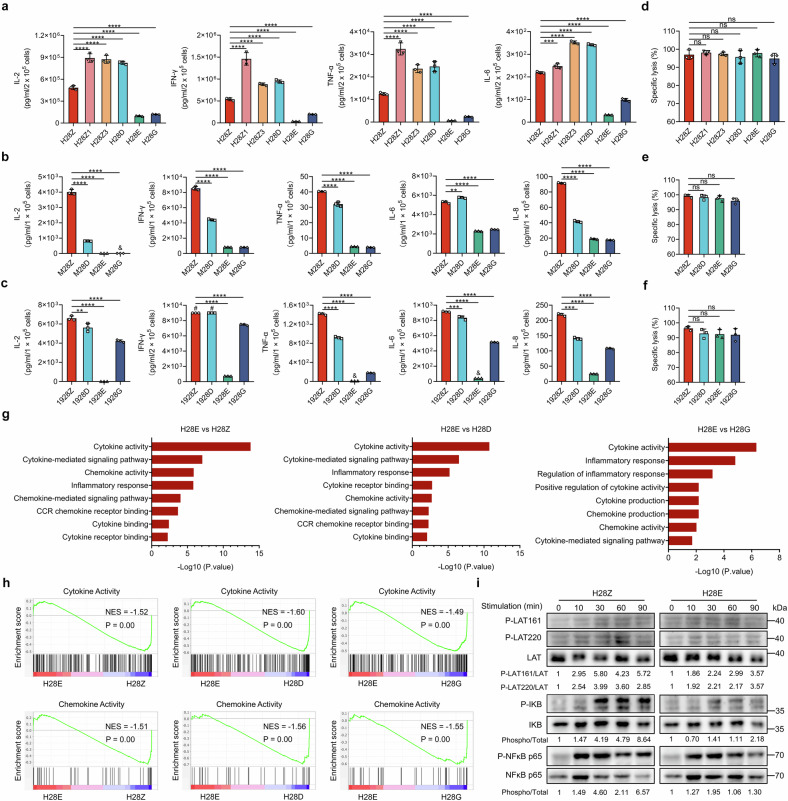
CD3ε-based CAR-T cells produce low levels of inflammatory cytokines. **a**–**c** T cells engineered with CARs containing varied ICDs were cocultured with the corresponding malignant cells overexpressing HER2 (**a**), mesothelin (**b**) or CD19 (**c**) (E:T = 1:1) for 24 h. The levels of indicated cytokines produced by CAR-T cells were measured and plotted. **d**–**f** CAR-T cells were cocultured with indicated malignant cells that expressed the corresponding antigens (E:T = 1:1) for 24 h, and the percentages of cell lysis were calculated and plotted. “19” in the CAR names refer to an scFv against CD19. **g**,**h** Normalized enrichment scores of significantly changed gene sets in H28E versus other groups of CAR-T cells as determined in Gene Ontology (**g**) analyses (*n* = 3 replicates per group). The enrichment of gene sets related to cytokine or chemokine activity between H28Z and other groups of CAR-T cells was also determined by GSEA (**h**; *n* = 3 replicates per group). **i** HER2-targeted CAR-T cells were stimulated by incubation with HER2-overexpressing PC-9 cells for indicated times. Cells were then harvested for Western blot analysis. The intensities of the protein bands were quantified by the ImageJ software, and the normalized band densities were indicated. Data are representative images and expressed as the means ± SD of three independent experiments. **P < 0.01, ***P < 0.001, ****P < 0.0001; # and &, concentrations beyond the measurable range; ns, non-significant

Excessive cytokine secretion poses a barrier to the beneficial outcome of anticancer therapy by CAR-T cells.^29^ We measured the levels of inflammatory cytokines released by CAR-T cells upon priming through coculture with malignant cells. Consistent with a previous report,^30^ we observed that H28Z1 and H28Z3 CAR-T cells secreted comparable or even more cytokines than the conventional CAR-T cells (Fig. 3a). By contrast, H28E and H28G CAR-T cells are featured by substantially reduced cytokine release when compared to H28Z CAR-T cells (Fig. 3a). In order to verify that substitution of an ICD from alternative CD3 chains for the canonical CD3ζ endodomain can mitigate cytokine production, we generated mesothelin- and CD19-targeted CAR-T cells, and co-cultured these cells with HeLa cells or a CD19-positive human leukemia cell line, Nalm6, respectively. As a result, we detected similarly declined cytokine release by the novel CAR-T cells compared with their conventional counterparts (Fig. 3b, c). The decline in cytokine release was in contrast to comparable cytolytic activities of the above CAR-T cells despite the ICD substitution (Fig. 3d–f). Since CARs with a CD3ε-derived ICD seemed to outperform others in reducing cytokine release, we compared the RNA-seq data of H28E with other CAR-T cells, and validated an enrichment of gene clusters for cytokine-related pathways like cytokine/receptor interaction, transcriptional regulation and chemokine activity (Fig. 3g, h). Linker for activation of T cells (LAT) is a key player in signal transduction of T cells, whose phosphorylation at tyrosine 161 and tyrosine 220 is closely linked to inflammatory cytokine synthesis and the cytolytic activity, respectively.^31^ Compared to the conventional CAR-T cells, H28E showed attenuated antigen-induced phosphorylation of LAT at the 161^st^ but not the 220^th^ tyrosine, and these new CAR-T cells exhibited a coordinately reduced activation of NF-κB pathway that orchestrates cytokine production (Fig. 3i).^32^ These data suggest that CD3ε-based CAR-T cells surpassed others in circumventing undesired cytokine release while maintaining sufficient cytolytic activities.

### Modest cytomembrane expression of CAR composed of a CD3ε ICD

**Fig. 4:**
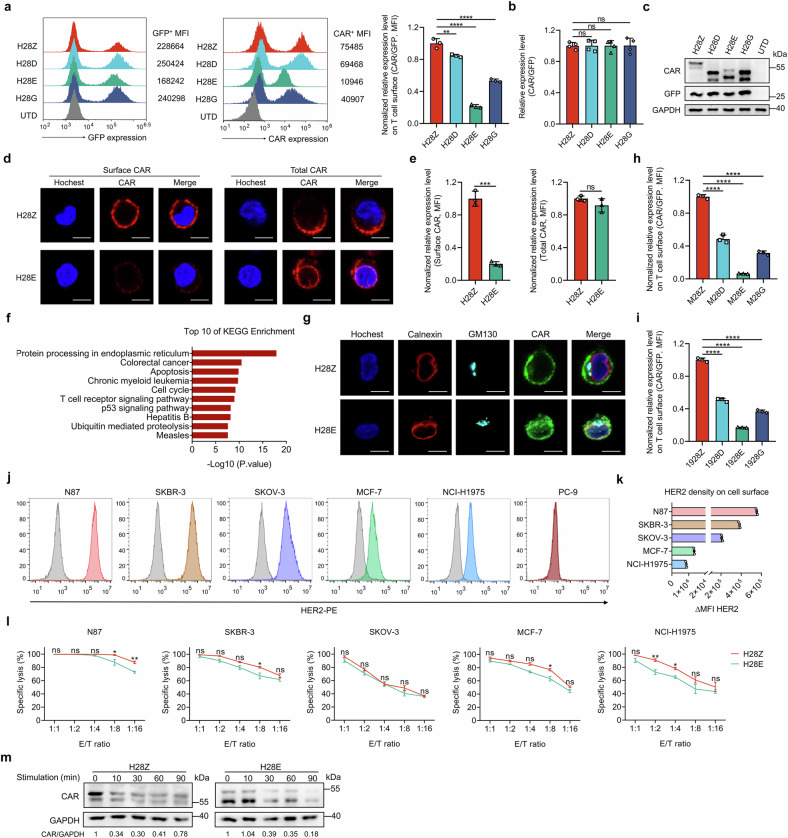
CARs with reconstituted ICDs display a low cytomembrane expression on T cells. **a** FCM assays for the cell surface levels of HER2-targeted CARs and for levels of GFP co-translated with CAR on engineered T cells. Levels of cytomembrane HER2-targeted CARs normalized to GFP were plotted. **b** Quantitative RT-PCR assay for HER2-targeted CARs in engineered T cells. **c** Western blot assay for HER2-targeted CARs in engineered T cells. **d** H28Z and H28E CARs were introduced into T cells and immunofluorescence staining was performed to show distinct expression patterns of these CARs. Bar, 10 μm. **e** Levels of cytomembrane and total HER2-targeted CARs were measured and plotted in which the levels of H28Z CAR were designated arbitrarily as 1. **f** Human primary T cells were lentivirally infected to express H28Z and H28E CARs, and cells were harvested 10 d later and subjected to RNA-Seq. KEGG pathway enrichment analyses of the differentially expressed genes were performed (*n* = 3 replicates per group). **g** Immunofluorescence staining for evaluation of subcellular co-localization of indicated CARs with the endoplasmic reticulum protein Calnexin or the Golgi apparatus marker GM-130. Cells were co-stained with Hoechst 33342 to visualize nuclear morphology. Bar, 10 μm. **h**, **i** Levels of cytomembrane mesothelin- or CD19-targeted CARs normalized to co-translated GFP were plotted. **j**, **k** HER2 expression on the surface of indicated cell lines were measured via FCM (**j**) and HER2 densities were plotted (**k**). **l** H28Z and H28E CAR-T cells were cocultured with indicated malignant cells at varied E/T ratios for 16 h, and the percentages of cell lysis were calculated and plotted. **m** HER2-targeted CAR-T cells were primed by coculture with HER2-overexpressing PC-9 cells for indicated times. Cell lysates were then prepared for Western blot analyses. Data are representative images and expressed as the means ± SD of three independent experiments. *P < 0.05, **P < 0.01, ***P < 0.001, ****P < 0.0001; ns, non-significant

CD3ε forms heterodimers with either δ or γ chain before the full assembly of TCR/CD3 and their exit from the ER.^33^ We thus examined whether CARs composed of different ICDs were equally transported through the ER-Golgi system to the cytomembrane. Intriguingly, CARs with an ICD from alternative CD3 chains, especially the one containing a CD3ε endodomain, showed remarkably lower expression on the cytomembrane as revealed by a ratio of CAR levels to those of co-translated GFP (Fig. 4a). In parallel, we failed to observe an apparent decline in on-membrane expression of CARs composed of a truncated CD3ζ signaling module (Supplementary Fig. 4a, b). The dramatic decrease in cytomembrane display was not due to inefficient transcription or translation of the coding gene of CD3ε-based CAR since its total level was comparable to that of the conventional CAR (Fig. 4b, c). Accordingly, immunofluorescence staining revealed an abundant cytoplasmic residency of the H28E CAR (Fig. 4d). Flow cytometry assay validated significantly reduced surface display of the H28E CAR although both H28E and H28Z CARs were abundantly expressed by the modified T cells (Fig. 4e). It was reported that the CD3 subunits, unless assembled into the TCR/CD3 complex, were distinctly localized in T cells.^34^ Indeed, we observed a predominant distribution of endogenous CD3ζ on cell surface, and localization of CD3ε both on the cytomembrane and in the ER of T cells (Supplementary Fig. 5a, b). RNA-seq was then performed using unstimulated CAR-T cells, and KEGG pathway analysis consistently suggested an enrichment of genes involved in protein processing in the ER (Fig. 4f). In line with these findings, we observed that H28E but not H28Z CAR displayed an apparent colocalization with the ER marker calnexin (Fig. 4g). The universality of distinct patterns of CAR expression was next explored. Compared with the canonical ζ chain-based CAR, we observed declined levels of cytomembrane ri-CARs that recognized mesothelin or CD19 (Fig. 4h, i). We then examined CAR expression on the surface of transduced cell lines. When introduced into Jurkat cells, ri-CARs showed a similarly lower cytomembrane expression than their conventional counterpart (Supplementary Fig. 6a). By contrast, we detected a marked decrease exclusively in the surface level of CD3ε-based CAR but not those of other ri-CARs when expressed in HEK293T cells (Supplementary Fig. 6b). These data suggest the involvement of endogenous ε chain in restraining the on-membrane display of ri-CARs. We next substituted the costimulatory domain of 4-1BB for the CD28 counterpart, and measured the surface expression of the resulting CARs in engineered T cells (Supplementary Fig. 6c). Although reduced surface display of ri-CARs was also observed, we failed to detect a dramatically lowered cytomembrane level of the CD3ε CAR compared with the conventional CAR (Supplementary Fig. 6d), which suggest that other intracellular regions might also play regulatory roles in CAR transport or cytomembrane anchoring. Together, these data suggest that the different CD3 domains incorporated in CARs might affect the subcellular distribution of CARs.

### CD3ε-based and conventional CAR-T cells display comparable tumor antigen thresholds required for cytolysis

We next investigated whether the decrease in cytomembrane levels of these novel CARs impairs their cytotoxicity to malignant cells. Human neoplastic cells that express different levels of HER2 were used as targets of CAR-T cells (Fig. 4j, k). We found that H28Z and H28E CAR-T cells are almost equivalent killers of these HER2-positive cells except in conditions with an extremely low antigen density or effector/target ratio (Fig. 4l). Similarly, these CAR-T cells displayed comparable cytotoxicities against PC-9 cells modified to express varied levels of HER2 (Supplementary Fig. 7a, b). We next sorted the transduced cells to obtain CAR-T cells with different surface densities of CARs for tumoricidal assays (Supplementary Fig. 8a, b). We observed that the cytolytic activities of the conventional H28Z CAR-T cells were attenuated following a decrease in surface CAR level, and these CAR-T cells exhibited a pronounced lower tumoricidal capability than H28E cells that expressed equivalent levels of surface CAR (Z-L v.s. E-WT) (Supplementary Fig. 8c), which implies that the low surface display of these CARs represents a unique self-limited property rather than at the expense of cytotoxicity. These findings were supported by the Western blotting assay showing that the total level of H28E CAR remained comparable to that of H28Z CAR during incubation with the target cells, suggesting that the intracellular CAR can be dynamically mobilized for cytolysis (Fig. 4m). Thus, CD3ε-based CAR displayed a restrained surface expression on transduced T cells without significantly improving the threshold of tumor antigen density required for efficient killing.

### Self-restrained surface CAR display is attributed to ER localization dependent upon the ICD conformation

**Fig. 5:**
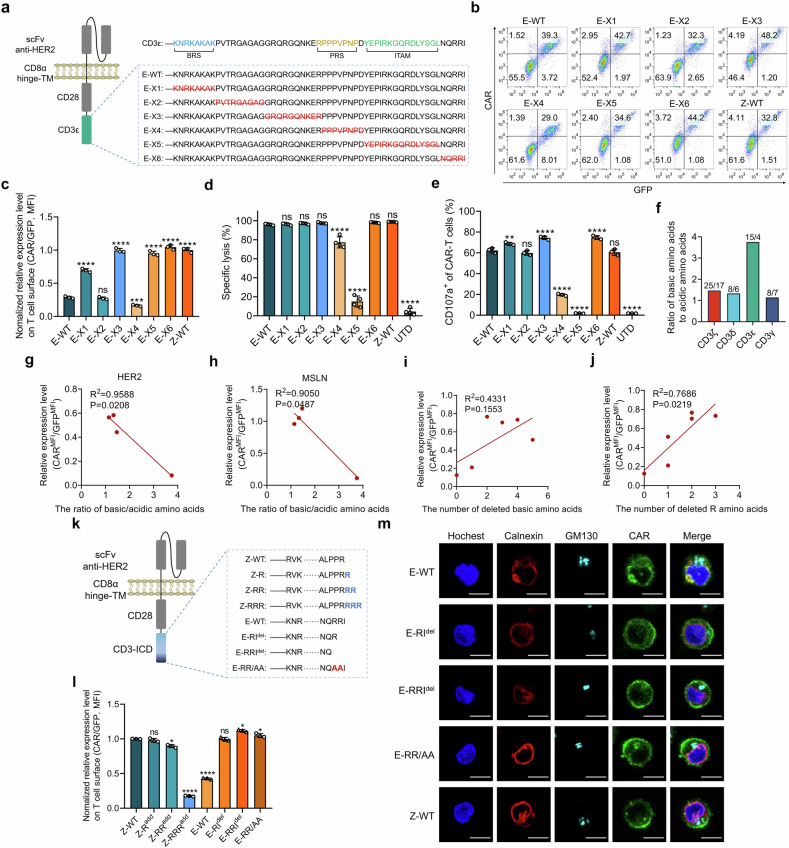
Surface expression of CD3ε-based CAR is related to C-terminal basic residues. **a** The segments of CD3ε endodomain were serially deleted from H28E CAR used for generation of CAR-T cells. **b**, **c** T cells engineered with the above intact or truncated CARs were subjected to FCM assays. The relative surface CAR levels were calculated by normalizing the MFI of CAR to that of GFP. **d**, **e** Cytolytic capabilities of CAR-T cells in (**a**) were examined by measuring the activities of luciferase constitutively expressed by the cocultured HER2-positive PC-9 cells (**d**), and degranulation of CAR-T cells were assayed by FCM assay for CD107a (**e**). **f** Numbers of basic and acidic amino acid residues in ICDs of different CD3 subunits were indicated, and the ratios of basic to acidic residues were plotted. **g**, **h** HEK293T cells were transduced to express CAR variants targeting HER2 or mesothelin and subjected to FCM assay for surface CAR levels. The correlation between surface CAR levels and the basic/acidic residue ratios in the ICDs of CARs was analyzed. **i**, **j** Illustrations showing the correlation between surface levels of the truncated CD3ε CARs and the numbers of deleted basic residues (**i**) or arginines (**j**) in the ICDs of CARs. **k**–**m** Variants of H28E CAR whose C-terminal residues were deleted and H28Z CAR with added C-terminal arginine residues were constructed (**k**). Surface expression of these CARs was measured via FCM after introduction into T cells (**l**). Cells were also subjected to immunofluorescence staining to detect the subcellular localization of these CARs (**m**). Bar, 10 μm. Data are representative images and expressed as the means ± SD of three independent experiments. *P < 0.05, **P < 0.01, ***P < 0.001, ****P < 0.0001; ns, non-significant, compared with E-WT (**c**–**e**) or Z-WT (**l**) group

We next tried to decipher the structure of CD3ε that potentially mediates the modest surface expression of H28E CARs in engineered T cells. CD3ε is known to promote TCR recycling and degradation via recruitment of the endocytic adaptor Numb.^35^ We thus asked whether Numb plays a role in reducing the cytomembrane anchoring of H28E CAR. However, amino acid mutation or deletion in the established Numb-interacting motif of CD3ε failed to affect the expression patterns of the resulting CARs (Supplementary Fig. 9a–c),^35^ suggesting alternate mechanisms underlying the incomplete surface display of these CARs. We then constructed CD3ε-based CARs with serially truncated endodomains (Fig. 5a), and found that deletion of several CD3ε segments exhibited the potential to significantly improve cytomembrane localization of CARs (Fig. 5b, c). As expected, the absence of ITAM or the proline-rich stretch (PRS), a motif required for TCR signaling through recruiting the adaptor protein Nck,^36^ resulted in loss or remarkably compromised cytolytic activities of CAR-T cells (Fig. 5d, e). These findings suggest the involvement of multiple sites in fine-tuning the cell surface expression of CARs consisting of a CD3ε ICD.

Previous studies reported wide electrostatic interactions between basic residues of the CD3 chains and acidic phospholipids in the inner leaflet of the biolayer membrane, which sequester key tyrosines from downstream kinases unless TCR ligation-elicited Ca^2+^ influx neutralizes the negatively charged phospholipids.^37^ We thus infer that these mechanisms may also contribute to the sequestering of CARs by intracellular membrane and shielding CARs from transporters in the organelle, and thus prevent them from transmembrane trafficking and cell surface display. In support of this hypothesis, we found that the CD3ε endodomain possessed the highest ratio of basic to acidic amino acids among all CD3 subunits (Fig. 5f; Supplementary Fig. 10), and the basic/acidic residue ratios in the ICD were inversely associated with surface expression of CARs in HEK293T cells (Fig. 5g, h). In agreement with these observations, we found that the numbers of deleted arginine residues among the above truncated CARs correlated well with the increase in the membrane levels of CARs (Fig. 5i, j). Conversely, inclusion of a C-terminal overhang of arginines in the H28Z CAR reduced its cytomembrane expression, whereas deletion of arginine(s) in the carboxyl tail of H28E or mutation to alanine improved their surface localization (Fig. 5k, l). While immunofluorescence staining revealed abundant ER colocalization of the H28E CAR (Fig. 5m, E-WT), a decrease in the intracellular storage and a concurrent upregulation in the cytomembrane level of CARs were observed when the C-terminal arginine was deleted (Fig. 5m, E-RI^del^), which was further aggravated by the absence of the C-terminal 2 arginines (Fig. 5m, E-RRI^del^ & E-RR/AA). These data suggest that ionic interactions between the ICD of CARs and the endomembrane system dictate CAR distribution in T cells before antigen cross-linking.

We next asked how the restrained surface expression of CARs affects the cytoxicity and cytokine release of primed CAR-T cells. While deletions of the C-tail basic residues from H28E didn’t remarkably change the cytotoxicity of CAR-T cells, these modifications significantly increased cytokine secretion by the primed CAR-T cells, respectively (Supplementary Fig. 11a, b). By contrast, although addition of a basic amino acid overhang to H28Z relieved cytokine production by the primed CAR-T cells, this resulted in a significant decrease in the cytolytic activity (Supplementary Fig. 11c, d). We next compared the cytokine release by antigen-primed H28E (E-WT) and H28Z (Z-WT) CAR-T cells, as well as those expressing a CD3ε-based CAR variant (E-X6) that exhibited very close surface expression with H28Z CAR (Fig. 5b, c). While the increase in cytomembrane expression of CAR resulted in elevated cytokine production (E-X6 v.s. E-WT), these H28E CAR-T cells still secreted significantly lower cytokines than H28Z cells (E-X6 v.s. Z-WT; Supplementary Fig. 12). These data suggest that CD3ε-based CAR-T cells outperformed others in circumventing excessive cytokine release, which is attributed coordinately to a modest CAR surface expression and the inherent property of the ICD in signal transduction.

### Superior performance of CD3ε-based CAR-T cells against in vivo tumors

**Fig. 6:**
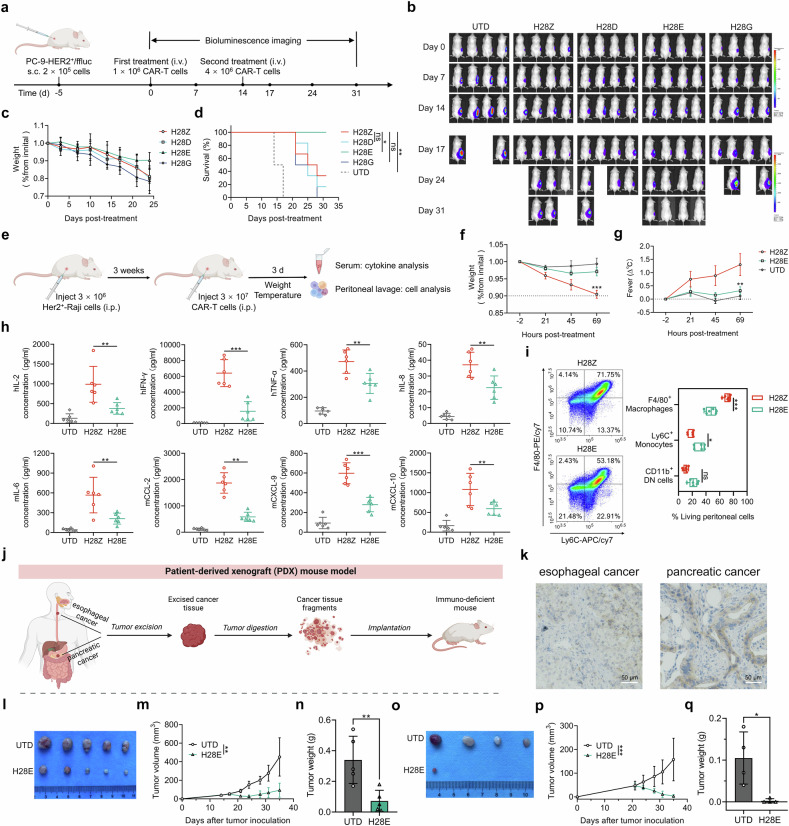
Suppression of in vivo tumor growth by T cells expressing conventional and novel CD3 domain CARs. **a**–**d** As shown in the schematic illustration for the time-line (**a**), B-NSG mice were challenged with tumor cells and received adoptive treatment with CAR-T cells on indicated days. Bioluminescent imaging for tumors was performed on indicated days after administration of CAR-T cells (**b**). Fold changes in mouse body weight after tumor inoculation were also monitored (**c**; *n* = 6), and survival of mice receiving treatment with CAR-T cells was recorded and plotted (**d**; *n* = 6). **e**–**i** In the in vivo cytokine release assay, SCID-beige mice were challenged with HER2-expressing lymphoma Raji cells and received adoptive treatment with CAR-T cells on indicated days (**e**). The samples of serum and peritoneal lavage were then collected for analysis. Changes in mouse body weight (**f**) and body temperature (**g**) were monitored. The levels of inflammatory cytokines in the serum were measured via FCM (**h**), and the ratios of indicated cell subsets in the peritoneal lavage was measured via FCM (**i**). **j**–**q** Clinical esophageal and pancreatic cancer samples were collected and used for generation of PDX models following the protocol shown in (**j**). Immunohistochemistry staining verified the expression of HER2 in tumor tissues (**k**). Mice bearing esophageal cancer PDX were treated with unmodified or H28E CAR-expressing T cells 14 days after tumor implantation, monitored for tumor growth and sacrificed 21 days post-treatment for isolation and weighing of the tumors (**l**–**n**; *n* = 5). Mice bearing pancreatic cancer PDX were treated 21 days after tumor implantation, monitored for tumor growth and sacrificed 14 days post-treatment for isolation and weighing of the tumors (**o**–**q**; *n* = 4). Illustrations in **a, e** and **j** were generated via an online tool (BioRender.com). *P < 0.05, **P < 0.01, ***P < 0.001; ns, non-significant

The antitumor efficacy of CAR-T cells was evaluated through intravenous administration on mice bearing HER2-positive xenograft tumors (Fig. 6a). While the initial infusion of all CAR-T cells accounted for similarly decreased tumor burden, the subsequent booster injection of a higher dose H28E CAR-T cells caused complete tumor elimination and strikingly improved animal survival, which were in contrast to tumor progression or animal death when treated with other groups of CAR-T cells (Fig. 6b). Meanwhile, we detected minimized influence of H28E CAR-T cells on body weight in comparison with other groups (Fig. 6c), and remarkably prolonged survival of treated animals (Fig. 6d). We next evaluated the anti-tumor persistence via prolonged monitoring of tumor regression after a single dose administration of CAR-T cells (Supplementary Fig. 13a), and detected that H28E but not the conventional H28Z CAR-T cells induced long-term disease remission (Supplementary Fig. 13b, c), and markedly improved survival of mice (Supplementary Fig. 13d). We then prepared splenocytes from the above mice receiving CAR-T cell therapy, and observed that H28E cells constituted a higher percentage of the splenocytes (Supplementary Fig. 13e) and consisted of a lower population of cells expressing PD-1 than H28Z cells (Supplementary Fig. 13f). To explore whether the ameliorated cytokine production also contributed to the superior performance of H28E cells, we established a documented CRS model through challenging of mice with human lymphoma Raji cells modified to express HER2 (Fig. 6e).^38^ We found that these mice receiving treatment with H28E cells exhibited substantially alleviated body weight loss or fever when compared to those infused with conventional H28Z cells (Fig. 6f, g). Consistently, we detected remarkably lower cytokine levels in the serum, probably due to reduced expansion of pro-inflammatory cell populations as detected in the peritoneal lavage of mice infused with H28E cells, compared with those treated with conventional CAR-T cells (Fig. 6h, i). Since C-terminal modification of the classical H28Z CAR also relieved cytokine synthesis by CAR-T cells, we next tested whether this led to improved antitumor performance in vivo (Supplementary Fig. 14a). Unfortunately, we found that the addition of a basic overhang to H28Z failed to boost the tumoricidal capability of CAR-T cells in vivo (Supplementary Fig. 14, b–d). Thus, H28E CAR-T cells outperform their conventional counterpart in tumor suppression, which is largely attributed to the unique property of the intracellular signaling module of CD3ε.

We then assessed the efficacy of CAR-T cells by employing tumor models that endogenously express HER2. Mouse xenograft tumors were generated using a human breast cancer cell line, SKBR-3, and mice were injected with CAR-T cells for treatment (Supplementary Fig. 15a). While administration of either group of CAR-T cells caused tumor repression, H28E but not H28Z cells effectively repressed tumor development after rechallenging of mice with neoplastic cells (Supplementary Fig. 15b, c). In a xenograft model of MCF-7 cells that expressed modest levels of HER2, administration of either group of CAR-T cells led to moderately but comparably retarded tumor growth, suggesting the non-inferiority of H28E compared with conventional CAR-T cells in combating malignancies expressing low-density tumor antigens (Supplementary Fig. 15d–f). HER2-positive patient-derived xenograft (PDX) tumor models were finally established using freshly collected esophageal and pancreatic specimens (Fig. 6j, k). We observed that treatment of these mice with the H28E CAR-T cells significantly suppressed tumor growth or led to complete tumor eradication (Fig. 6l–q). Collectively, these data suggest that CARs comprising a CD3ε endodomain are superior to other CARs in conferring persistent tumoricidal activity and improved safety on engineered T cells.

## Discussion

Personalized CAR-T cell therapy has arisen as an effective approach for cancer treatment despite the challenges it confronts in solid tumor infiltration and inflammatory cytokine release.^39^ While conventional CARs consist of the extracellular antigen recognition moiety, the transmembrane and costimulatory domains from natural immune proteins, and an intracellular signaling domain (ICD) normally from CD3ζ, recent studies have disclosed the possibility of further improving the architecture of CARs without impairing the anti-tumor efficacy.^18,40^ Here, we optimized the configuration of CARs by replacing the entire ICD of CD3ζ, which possesses totally 3 ITAMs, with the one ITAM-containing endodomain of different CD3 subunits.^13^ We established that the resultant CAR-T cells displayed antigen-specific cytolytic activities comparable to the conventional CAR-T cells, and showed lowered tonic signaling, substantially ameliorated exhaustion, and improved capacity of differentiation into memory subsets. These findings are in agreement with a recent report that substitution of the ICD from alternative CD3 subunits for the ζ chain-derived domain conferred on T cells comparable antigen-specific cytotoxicity and in vivo tumor-suppressive activity.^19^ More importantly, CAR reconstitution with the endodomain from CD3ε yielded competent CAR-T cells with self-restrained on-membrane CAR expression, which contributed to remarkably decreased cytokine production (Supplementary Fig. 16). However, although our mechanistic study implicated the involvement of carboxyl-terminal basic residues in determining the expression patterns of CARs, additional investigations are required to probe the conformational basis of CARs for the superiority of CD3ε endodomain in terms of the accessible or sequestered sites in the structure and the 3-dimensional contact with the biolayer membrane or intracellular transporters.

In the current study, we demonstrated that an endodomain encompassing a single ITAM from any of the CD3 chains can be independently harnessed as the intracellular signaling module for CARs that target solid tumor cells, which is in contrast to the predominant inclusion of a CD3ζ domain in current CAR-T cell therapy mainly against hematopoietic malignancies.^2^ Antigen engagement of TCR has been documented to induce T cell activation via complicated signal pathways elicited by programmed recruitment of multiple kinases to the TCR/CD3 complex.^13^ By contrast, how CAR-T cells are primed upon encounter of neoplastic cells remains largely elusive.^2,12^ In addition, tonic signal has been widely accepted as a chief driver of CAR-T cell exhaustion that leads to compromised anti-tumor persistence.^7,8^ However, it is unclear how the ICD configuration of CARs affects the intensity of tonic signal. We found that compared with conventional CD3ζ-based CARs, those with a single ITAM in the ICD generated significantly reduced tonic signal, alleviated activation-induced apoptosis and enhanced proportions of cell subsets with memory phenotype. Nonetheless, T cells expressing the reconstituted CARs varied in tumoricidal potency and cytokine release after priming, suggesting that the diversity of other ICD segments or the characteristics of individual CD3 subunits might also play essential roles in tailoring the activating signal of CAR-T cells.^14,33^ Thus, our findings necessarily reflect the combinatorial corollary of an ITAM multiplicity model, in which reduced ITAM(s) attenuates the downstream signal intensity, and a differential signaling model, in which the signaling modules in different CD3 chains determine the downstream molecular events responsible for distinct phenotypes of CAR-T cells.^41,42^

It remains so far an issue of debate whether the release of cytolytic granules and the secretion of cytokines by CAR-T cells are separately controlled or cross-regulated by intracellular signal pathways.^43^ To this end, we observed significantly reduced phosphorylation of LAT at tyrosine 161 (Y132 for the mature protein after removal of the signal peptide) in primed T cells engineered with reconstituted CARs compared with the conventional CAR-T cells, suggesting CD3ζ is probably required for LAT phosphorylation at this site during T cell receptor signaling. However, while LAT Y161 phosphorylation has been documented to license cytokine synthesis via PLCγ/IKK/NF-κB pathway,^22^ we detected attenuated activation of NF-κB and consequently low levels of cytokine production exclusively in CD3ε-based CAR-T cells, highlighting the possibility of certain aptitudes of the ε chain that control signal transfer from the proximal T cell signalosome to downstream molecules, e.g. a unique time course of LAT phosphorylation. In addition, cytokine release by antigen-primed CAR-T cells is controlled by ICD-derived signaling such as recruitment of the inhibitory kinase Csk or Nck in case of CD3ε.^15,44^ Thus, our investigations cannot rule out the possibility that the proximal kinases or phosphatases recruited by CD3ε also contribute to the regulation of cytokine production by CAR-T cells.^45,46^

Cytokine production by CAR-T cells has been reported to require a much higher threshold of antigen and receptor densities than their cytolytic activity.^47^ Consistently, we identified a low on-membrane expression of CD3ε CAR albeit it is sufficient to elicit cytolysis upon antigen engagement. Our observations are reminiscent of a recent report using alternative CD3 subunits as the sources of a CAR ICD.^19^ In that report, Velasco Cárdenas et al. detected a similar trend of mitigated cytokine release by these novel CAR-T cells, and observed that CD3ε-based CAR outperformed others in antigen-induced T cell activation, although they didn’t address the surface expression of these CARs.^19^ Despite these consistent findings, we demonstrated that, of the 3 novel configuration CARs, CD3ε-based CAR is superior in minimizing inflammatory cytokine release rather than antigen-specific priming. Since the above study employed 4-1BB and we used CD28 domains for CAR design, these discrepancies suggest that the costimulatory domain and the CD3-derived signal module of the CAR might synergistically determine cytokine release by primed CAR-T cells.^19^ However, the density of available antigens also plays a pivotal role in fine-tuning CAR-T cell activation.^47^ While the 4-1BB costimulatory domain usually evokes a relatively mild response of CAR-T cells, CD28 might serve as a more suitable derivation of costimulatory domain for CARs against low density antigens like those expressed by many solid tumors.^47,48^ Another issue that requires attention is the combination of tumor antigen and antigen recognizing scFvs, which might endow CAR-T cells with varied antitumor performance including the cytokine release feature. Indeed, we detected apparent differences in antigen-induced cytokine release between the novel CAR-T cells targeting HER2, mesothelin and CD19, suggesting that the affinity or additional interaction properties between scFv and the tumor antigen collaborate with other moieties of the CAR to determine the threshold of intracellular signaling that induces CAR-T cell activation.^49^

Electrostatic interaction with the biomembrane has been documented to intensively affect protein localization.^37,50,51^ In particular, the ionic interactions between basic ICD residues of CD3ε and acidic phospholipids facilitate its attachment to the inner leaflet of the cytomembrane and the insertion of the ITAM residues into the hydrophobic core of the lipid bilayer.^37^ We thus assumed that the uniquely high ratio of basic to acidic amino acids in the endodomain of the ε subunit mandates CAR association with phospholipids enriched in the intracellular membranes, which might sequester CARs, reduce their accessibility to transporters and intercept their cytomembrane localization.^37,52^ The unstructured positioning of CD3ε-based CAR is probably disrupted by calcium influx in the early antigen response, which triggers their cytomembrane trafficking and transmembrane reorientation required for adequate T cell activation and tumor repression.^37^ Thus, the relatively low density of cytomembrane CARs might underlie the insufficiency of intracellular signaling that transcriptionally activates cytokine genes.^47^ Nonetheless, the ensuing mobilization of intracellular CARs warrants effective cytolysis by these CAR-T cells. Therefore, these observations have implications for further optimizing the configuration of either an ε or ζ chain-based CAR probably via simple modulation of specific residues in the intracellular domain. In addition, it is noteworthy that unlike endogenous TCR genes that are expressed from their tightly controlled promoters in normal T cells, CARs are constitutively synthesized in engineered T cells, underlining the necessity of future exploration on the kinetics of surface and intracellular CARs orchestrated by endocytosis and subsequent proteasomal degradation.^2^ Collectively, our findings together with recent attempts to promote tumor infiltration of infused T cells provide beneficial approaches to improving the applicability of CAR-T cells in combating diverse malignancies.^53,54^

## Materials and methods

### Ethics statement

The animal experiments were approved by the Animal Experiment Administration Committee of the Fourth Military Medical University (Protocol ID: 20210565). All patients gave informed consent for collection of human tumor specimens. Collection of specimens from patients or peripheral blood from healthy donors followed a protocol approved by the Ethics Committee of Fourth Military Medical University (Xi’an, China; protocol ID: KY20213037-1).

### Cell lines and culture conditions

Human lung carcinoma PC-9 cells and T lymphoma Jurkat cells were purchased from Cell Bank of Chinese Academy of Sciences (Shanghai, China). Human cell lines including cervical cancer HeLa cells, leukemia Nalm6 cells, lung carcinoma NCI-H1975 cells, breast cancer SKBR-3 and MCF-7 cells, ovarian cancer SKOV-3 cells, gastric carcinoma N87 cells and human embryonic kidney (HEK) 293 T cells were purchased from American Type Culture Collection (ATCC, Manassas, USA). HEK293T cells were cultured in DMEM medium (Gibco, ThermoFisher Scientific, Gaithersburg, USA) with 10% FBS, 4 mM L-glutamine and 100 U/ml penicillin/streptomycin. HeLa cells were cultured in MEM media, SKBR-3 and SKOV-3 cells were maintained in McCoy’s 5a medium, MCF-7 cells were cultured in ATCC-formulated Eagle’s Minimum Essential Medium supplemented with 1.4 mL human recombinant Insulin (Gibco), and other cell lines were cultured in completed RPMI1640 medium (Gibco) supplemented with 10% FBS (Gibco) and 100 U/ml penicillin/streptomycin. PC-9 cells were stably transduced to co-express HER2 and firefly luciferase (HER2/Luc), while Nalm6 cells were transduced to co-express blue fluorescence protein and firefly luciferase. HeLa cells were transduced to express a firefly luciferase protein.

### Constructs of chimeric antigen receptors (CARs)

The second-generation CARs comprise a CD8a leader peptide, a single-chain variable fragment (scFv) specific for HER2 (P1h2),^55^ CD19 (FMC63),^56^ or mesothelin (SS scFv),^57^ followed by a CD8α transmembrane and hinge domain, a CD28 or 4-1BB co-stimulatory domain and an endodomain from the ζ, δ, ε or γ chain of CD3. CARs with a truncated CD3ζ endodomain that consisted of 1 of the 3 ITAMs were also generated. The CAR expression cassette was fused to a T2A sequence to achieve co-translation with green fluorescence protein (GFP). The expression cassettes for CARs with amino acid deletion, substitution or added C-terminal residue(s) in the endodomain were obtained via PCR technology and the use of a point mutation kit (Transgen, Beijing, China). For immunofluorescence or immunocytochemistry assays, the coding sequence of a Flag tag was inserted prior to that of the scFv for detection of CAR expression. All cassettes of CARs were cloned into an pLVX-EF1α-IRES-Puro lentiviral vector.

### Lentivirus production and transduction of human T cells

Replication-defective lentiviral vectors were introduced into HEK293T cells for packaging, and virus-containing supernatant was harvested, centrifuged to remove cell debris and stored at −80 °C until use. PBMCs were obtained from human healthy donors. Human T cells were isolated using the MojoSort Human CD3 T Cell Isolation Kit (Biolegend, San Diego, USA). Primary T cells were expanded by incubation with CD3/CD28 dynabeads (Gibco) at a T cell to bead ratio of 1:1, and cultured in X-VIVO15 medium (Lonza, Bend, USA) with 10% FBS and 100 IU/ml recombinant IL-2 (PeproTech, Rocky Hill, USA). Twenty-four hours after stimulation, T cells were incubated with lentiviruses for 24 h. At day 5 after stimulation, the dynabeads were removed, T cells were cultured continuously, and medium and IL-2 were changed every second day.

### Flow cytometry (FCM)

Cells were harvested, washed and resuspended in 100 μl PBS supplemented with 2% FBS, and incubated with antibody for 30 min at 4 °C. The following antibodies from Biolegend were used for detection of cell surface proteins: CD3-FITC, CD4-APC, CD4-PE, CD8-APC, CD8-PE, CD45RA-PE, CD45RA-BV650, CD62L-APC/Cy7, CD62L-BV421, PD-1-PE, TIM-3-APC/Cy7, LAG-3-APC, LAG-3-BV421, Annexin V-APC, Annexin V-PE/Cy7, CD25-PE, CD69-APC, CD11b-APC, F4/80-PE/Cy7, Ly6C-APC/Cy7, CD86-PE and Flag-PE/Cy7. The mouse IgG1 kappa isotype control antibody was purchased from eBioscience (ThermoFisher). CARs were detected by incubation with the recombinant human biotinylated HER2-Fc, CD19-Fc or Mesothelin-Fc protein (R&D Systems, Minneapolis, USA), and then with an APC or PE-cy5/streptavidin antibody (Biolegend). T cell subpopulations expressing varied levels of CARs (CAR^high^, CAR^medium^ and CAR^low^ T cells) were sorted by a Beckman Moflo Astrios EQ (Beckman, Brea, USA) according to the fluorescence intensity (FI) after staining of cells with recombinant HER2-biotin and the APC-streptavidin conjugate. BFP-expressing Nalm6 cells were identified through the Pacific Blue channel. For detection of total Flag-conjugated CAR, cells were treated with Cell Fixation & Cell Permeabilization Kit (BD Bioscience, Heidelberg, Germany), and stained with an anti-Flag antibody (Biolegend) for FCM assay.

### Cytotoxicity assays

For cytotoxicity assays of CAR-T cells to Nalm6 cells, 1.5 × 10^5^ BFP-positive neoplastic cells as targets were cocultured with CAR-positive or untransduced T cells at various ratios in X-Vivo15 medium in a 48 well plate. The cytotoxicity index was obtained by dividing the total target cells by the initial target cells used for co-incubation with effector cells. CAR-T or mock-transduced T cells were co-cultured with 2 × 10^4^ tumor cells expressing luciferase in X-Vivo15 medium in black-walled 96-well plates. Sixteen hours later, T cells and lysed/dead tumor cells were discarded. Wells were washed twice with D-PBS, followed by addition of 100 μl luciferase substrate to each well. Luminescence was measured in a luminescence plate reader. Killing efficiency was calculated as [1 − (RLUsample)/(RLUmax)] × 100, where RLU means relative light units. To visualize cell lysis, PC-9 cells overexpressing HER2 were incubated with Dil (Beyotime, Shanghai, China) in dark for 20 min, co-cultured with CAR-T cells (E:T = 2:1) for 6 h and then observed via fluorescence microscopy. To detect T cell degranulation and cytotoxicity, CAR-T cells were stimulated with target cells (1:1) in the presence of a Golgi Plug protein transport inhibitor (BD Biosciences, Franklin Lake, USA) for 6 h. For CD107a staining, PE-CD107a antibody (Biolegend) was added to the cocultured cells before FCM assay. For intracellular protein detection, CAR-T cells were harvested, fixed and permeabilized with Cell Fixation & Cell Permeabilization Kit (BD Bioscience) before FCM assays. Intracellular cytokine detection was performed via FCM using Alexa Fluor® 647-labeled Granzyme B (Biolegend, Cat. #372220) and PE-conjugated perforin antibody (Biolegend, Cat. #308106).

### Tonic signal evaluation

Cells were infected with recombinant lentiviruses to express different CARs, and then stained with an APC-conjugated CD69 antibody. FCM was performed with gated GFP-positive cells, and the MFI of CD69-APC and GFP were measured. CAR tonic signal index was calculated by dividing the MFI of CD69 by that of GFP.

### Immunofluorescence

To detect the surface expression of CARs, cells were harvested and stained with a Flag antibody (Proteintech, Rosemont, USA, Cat. #CL488-80010). When staining for intracellular protein, cells were permeabilized as aforementioned. Staining was performed with CoraLite® Plus 647-conjugated anti-Calnexin (Proteintech, Cat. #CL647-10427), CoraLite®555-conjugated anti-GM130 (Proteintech, Cat. #CL555-11308), or CoraLite® Plus 488-conjugated anti-Flag (Proteintech, Cat. #CL488-80010), FITC-conjugated anti-human CD247 (CD3ζ) (Biolegend, Cat. #644104), and FITC-conjugated anti-human CD3ε (Biolegend, Cat. #317306) antibodies. Cell nuclei were subsequently co-stained with Hoechst 33342 (Cell Signaling Technology [CST], Boston, USA, Cat. #4082S). Images were captured by a Nikon Ti2-E CSU-W1 microscope.

### Cytokine production measurement

CAR-T cells were washed with PBS, and resuspended in X-Vivo15 medium without exogenous cytokines. Prepared CAR-T cells were cocultured with tumor cells (1:1) in 96-well plates for 24 h, followed by collection of the supernatants and cytokine measurement via Luminex bead-based multiplex immunoassay (Bio-Techne, Minneapolis, USA) or LEGENplex^TM^ Human Essential Immune Response Panel (Biolegend, Cat. #740930) according to the manufacturers’ instruction.

### Western blotting

Ten days after expansion, cells were washed with PBS, and total protein was isolated after stimulation with target cells (E:T = 1:1). Blotting was performed under denatured conditions using the following antibodies: anti-LCK (CST, Cat. #2787 T), anti-ZAP70 (CST, Cat. #3165 T), anti-LAT (CST, Cat. #45533S), anti-ERK1/2 (Proteintech, Cat. #11257-1-AP), anti-AKT (Proteintech, Cat. # 60203-2-Ig), anti-IκBα (Proteintech, Cat. #10268-1-AP), anti-NFκB p65 (Proteintech, Cat. #80979-1-RR), anti-CD28 (CST, Cat. #38774S), and anti-GFP (CST, Cat. #2955S) antibodies for detection of the corresponding total protein, and anti-pLCK (CST, Cat. #2751 T), anti-pZAP70 (CST, Cat. #2701 T), anti-pLAT^161^ (Affinity Biosciences, Changzhou, China, Cat. #AF8200), anti-pLAT^220^ (CST, Cat. #3584 T), anti-pERK1/2 (Proteintech, Cat. #80031-1-RR), anti-pAKT (Proteintech, Cat. #66444-1-Ig), anti-pIκBα (Proteintech, Cat. #82349-1-RR), and anti-pNF-κB p65 (Proteintech, Cat. #82335-1-RR) antibodies for detection of the phosphorylation level of specific proteins. Blotting using an antibody against GAPDH (CST, Cat. #5174S) was included as a loading control and for normalization.

### Real-time PCR

CAR-T cells were separated by FACS, followed by total RNA extraction and cDNA synthesis using a reverse transcription kit (TaKaRa, Kusatsu, Japan). PCR reactions were performed with SYBR Green Mix (TaKaRa). Gene expression was normalized to β-actin or GFP.

### RNA sequencing

CAR-T cells (3 × 10^6^) were incubated with irradiated target cells (1:1) in a T-75 flask for 36 h. Cells were then separated by FACS (GFP-gated), washed and resuspended in 1 ml RNAiso Plus Reagent (TaKaRa). RNA was extracted and measured for quantity, purity and integrity. After PolyA capturation and cDNA library preparation, sequencing was performed using illumine Novaseq^TM^ 6000 according to the vendor’s protocol. Differentially expressed mRNAs were identified through a parametric F test comparing nested linear models (*P* < 0.05) via the R package edgeR. GSEA was performed using KEGG pathway and Gene Ontology terms.

### Cytotoxicity assessment using PDOs

Tumor tissues were collected from ovarian cancer patients undergoing surgical resection. The tissues were validated for HER2 expression via immunohistochemistry, followed by dissection of tissues and preparation of cell suspensions. These cells were then used for development of PDOs and measurement of the cytolytic activity of CAR-T cells as described.^58^ CAR-T cells were labeled with CytoTell™ Red650 (AAT Bioquest, Cat. #22255) prior to coculture, and the apoptosis of tumor cells was monitored through staining with CellEvent™ Caspase-3/7 Green (Thermo Fisher Scientific, Cat. #C10423).

### Tumor repression evaluation in xenograft mouse models

Six- to eight-week-old male NOD/SCID/IL-2Rγ-null (B-NSG) mice (Cat. #110586, Biocytogen, Beijing, China) were inoculated with HER2/Luc-expressing PC-9 cells via subcutaneously (s.c.) injection into the right flank, and were injected via tail vein with CAR-T or mock T cells for treatment. For generation of xenograft tumor models using other cell lines, mice were inoculated s.c. in the right flank with 5 × 10^5^ SKBR-3 cells or in situ in the fat pad of mammary gland with 5 × 10^5^ MCF-7 cells, followed by injection via tail vein with CAR-T or mock T cells. Mice were monitored for tumor development and obvious toxicity, and were humanely euthanized when tumors exceeded 2 cm in either direction. Tumor growth was examined by bioluminescent imaging with the Xenogen IVIS Lumina (Caliper Life Science), and the acquired imaging datasets were analyzed with the Living Image software (PerkinElmer, Waltham, USA). For evaluation of tumor regression by CAR-T cells in PDX models, HER2-positive tumor samples from esophageal cancer or pancreatic cancer patients were cut into small tissue fragments (about 1 mm^3^), and engrafted in B-NSG mice for passage through generations. The PDX samples were next s.c. transplanted in the right flank of B-NSG mice for propagation, and these mice were then subjected to intravenous treatment with 5 × 10^6^ cells. Tumor growth was monitored and mice were sacrificed for isolation and weighing of tumors 5 weeks after tumor engraftment.

### Evaluation of CRS in a mouse model

Six- to eight-week-old female CB17.Cg-PrkdcscidLystbg-J/Crl (SCID-beige) mice (Charles River) were intraperitoneally (i.p.) injected with 3 × 10^6^ HER2^+^ Raji-FLuc cells. After three weeks, mice were administered i.p. with 3 × 10^7^ CAR-T or mock T cells. To assess CRS toxicities, mice were monitored for daily activity, temperature and weight loss. Mice were humanely euthanized after three days of treatment, and the serum and peritoneal fluid were collected for measurement of cytokine levels and analysis of cell population composition, respectively.

### Statistical analysis

Statistical analyses were conducted using the Prism 8 (GraphPad) software. Data with two groups or more than two groups were analyzed using the two-tailed Student’s *t* test or one-way/two-way ANOVA, respectively. The statistical significance of Kaplan-Meier survival curves was assessed by the log-rank Mantel-Cox test. *P* < 0.05 was considered to be statistically significant.

## Supplementary information

Supplementary Figures

## Data Availability

All raw data generated by RNA-Seq are being deposited to the Gene Expression Omnibus database (GEO, accession #: GSE281230). Additional information to be used for reanalyzing the data reported in this paper will be available from the correspondence authors upon reasonable request.
