# Supplementary material for: Chimeric antigen receptor with novel intracellular modules improves antitumor performance of T cells
Source: Signal Transduct Target Ther. 2025 Jan 15;10:20. doi: 10.1038/s41392-024-02096-5 (PMC11733243; doi:10.1038/s41392-024-02096-5)
Supplement: Supplementary file 1 — Supplementary Figures [file 41392_2024_2096_MOESM1_ESM.docx]

Supplementary Materials for

Chimeric antigen receptor with novel intracellular modules improves antitumor performance of T cells

Pengju Wang^1,2,3,#^, Yiyi Wang^1,2,#^, Xiaojuan Zhao^1,#^, Rui Zheng^1,3^, Yiting Zhang^1^, Ruotong Meng^1^, Hao Dong^1,2^, Sixin Liang^1,2^, Xinyi He^1^, Yang Song^4^, Haichuan Su^4^, Bo Yan^1*^, An-Gang Yang^2,3,4*^, Lintao Jia^1*^

Correspondence to: [yanbo5870@fmmu.edu.cn](mailto:yanbo5870@fmmu.edu.cn); [agyang@fmmu.edu.cn](mailto:agyang@fmmu.edu.cn); jialth[@fmmu.edu.cn](mailto:yanbo5870@fmmu.edu.cn)

**This PDF file includes:**

Supplementary Figures 1 to 16

**
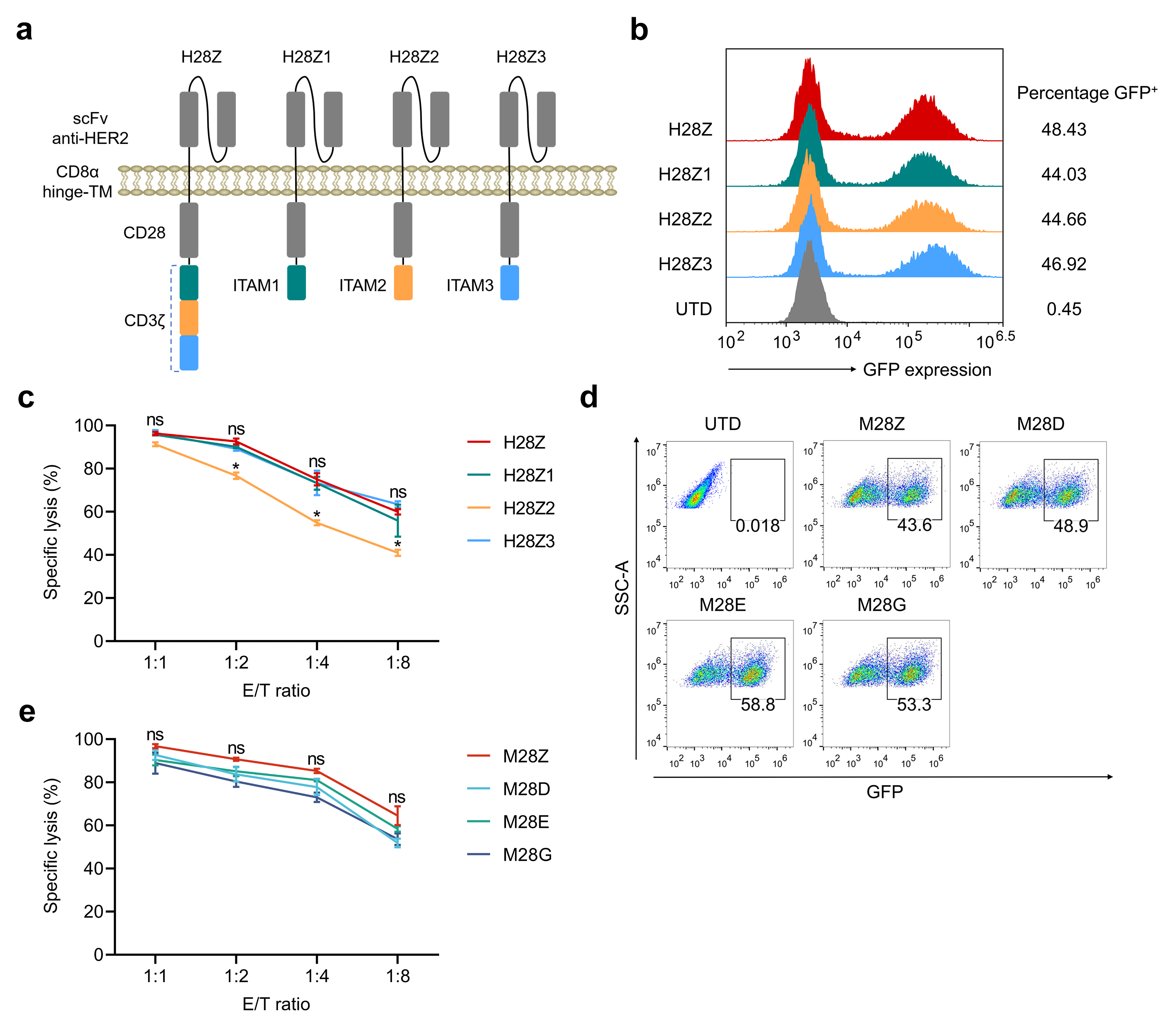
**

**Supplementary Fig. 1** Cytolytic activities of T cells expressing CARs with intact and truncated CD3 endodomains. **a** Schematic diagram of CARs comprising a HER2 scFv and a ζ chain endodomain that encompasses different ITAM(s). **b** Human PBMCs were infected with recombinant lentiviruses for indicated CARs, followed by FCM assay for CAR-expressing cells. **c** CAR-T cells were cocultured with PC-9 cells modified to overexpress HER2 at indicated E/T ratios for 16 h. The percentages of cell lysis were calculated and plotted. **d**, **e** Human PBMCs were infected with recombinant lentiviruses for indicated mesothelin-targeted CARs, followed by FCM assay for CAR-expressing cells (**d**). Cells were then cocultured with human cervical cancer HeLa cells at indicated E/T ratios for 16 h, and the percentages of cell lysis were calculated and plotted (**e**). “M” in the CAR names refer to an scFv against mesothelin. Data are representative images and expressed as the means ± SD of three independent experiments. **P* < 0.05; ns, non-significant, compared with H28Z CAR-T cells.

**
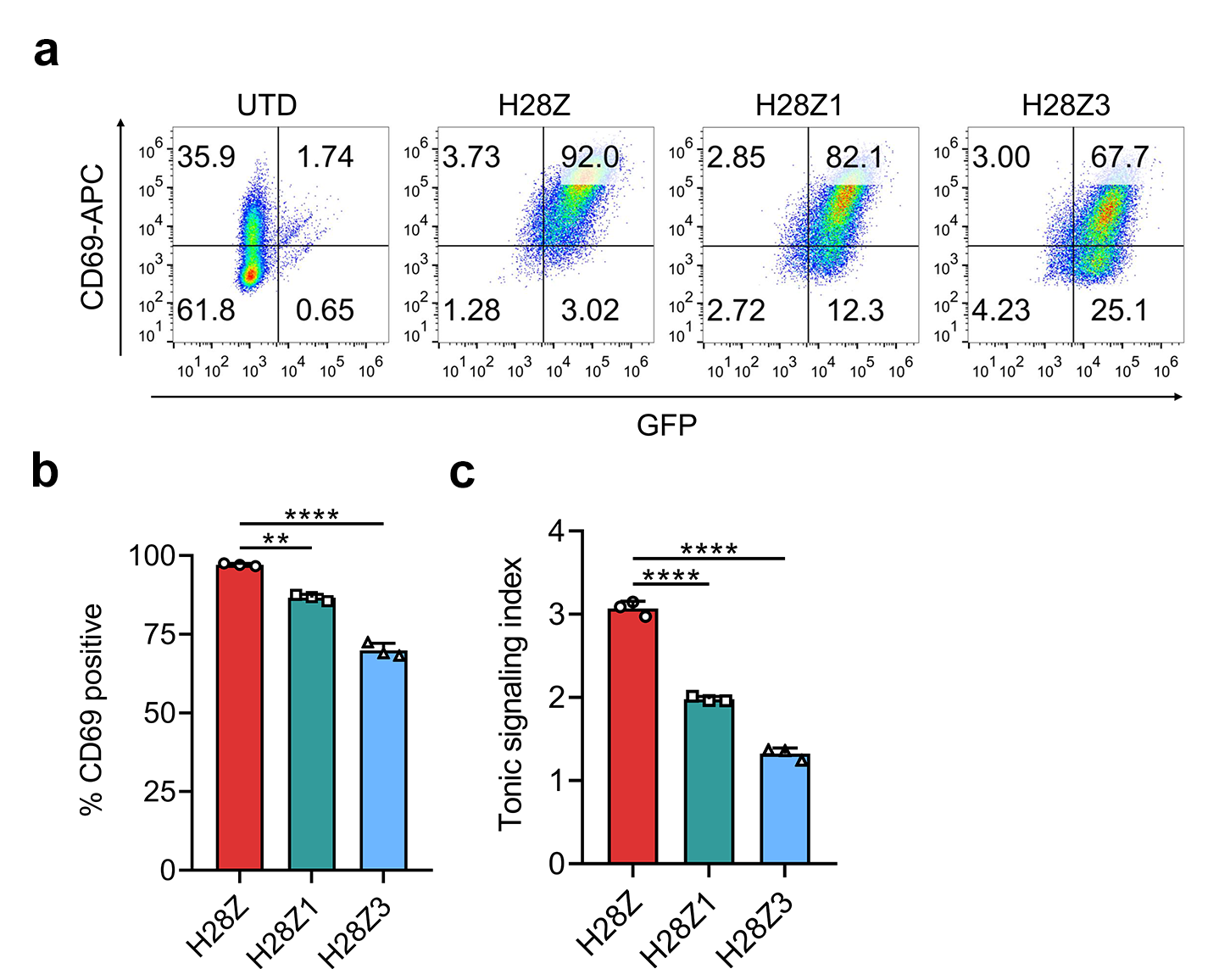
**

**Supplementary Fig. 2** CARs with a single ITAM of CD3ζ generate lower tonic signal than a conventional CAR. **a**-**c** CD69 levels on Jurkat cells transduced to express the indicated CARs were measured via FCM (**a**). The percentages of CD69-positive cells (**b**) and the tonic signal index **(c)** in each group of CAR-T cells were plotted. Data are representative images and expressed as the means ± SD of three independent experiments. ***P* < 0.01, *****P* < 0.0001.


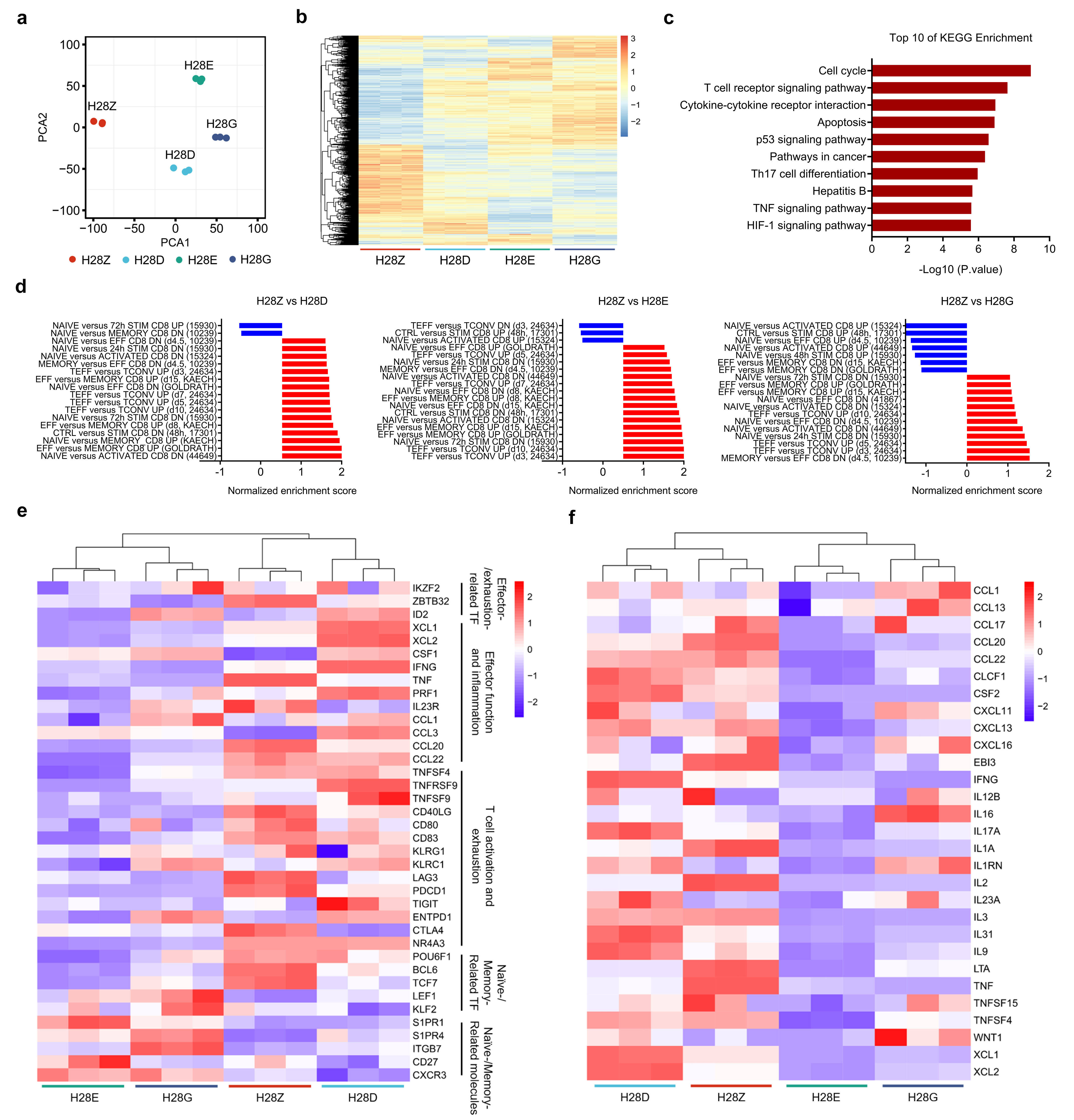


**Supplementary Fig. 3** Gene expression profiles in T cells expressing CARs with varied CD3 endodomains. **a**-**c** CAR-T cells were primed by coculture with HER2-overexpressing PC-9 cells for 36 h, and cells were harvested and subjected to RNA-Seq. A principal component analysis (PCA) for the obtained data (**a**), a heatmap of differentially expressed genes (DEGs, FDR *q* < 0.05) was shown (**b**), and a KEGG pathway enrichment analysis of the DEGs (FDR *q* < 0.05) were performed (**c**, n = 3 replicates per group). **d** Normalized enrichment scores of significantly upregulated or downregulated gene sets in H28Z versus other groups of CAR-T cells (n = 3 replicates per group) as determined by GSEA using the MSigDB C7 gene ontology sets. For all pathways, the false discovery rate (FDR q ≤ 0.25). GSE datasets are indicated in parentheses. STIM, stimulated; CONV, conventional. DN, downregulated. **e**, **f** Heatmaps for DEGs (FDR *q* < 0.05) involved in T cell behaviors (**e**) or cytokine production/signaling (**f**) was also shown (n = 3 replicates per group).


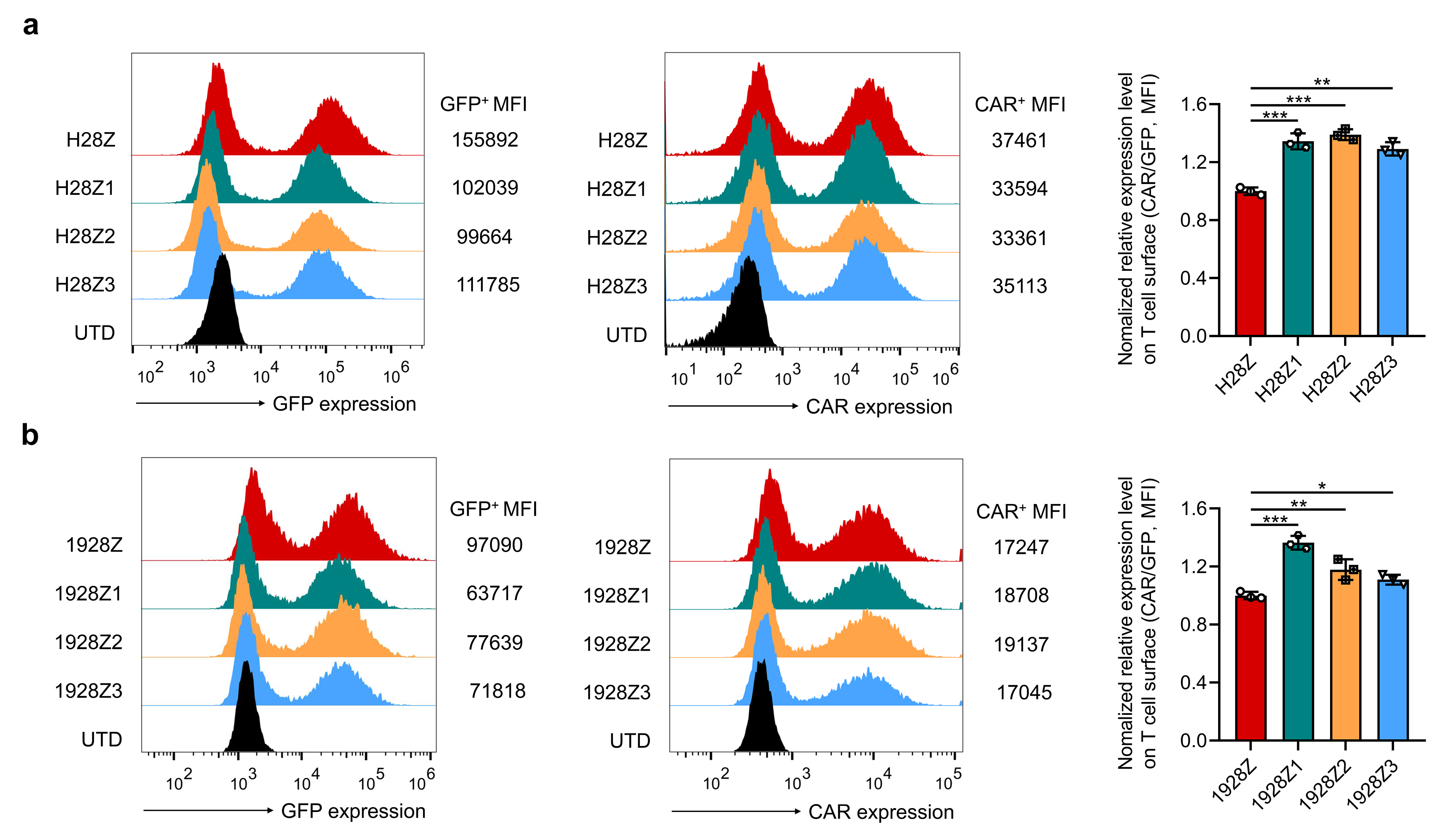


**Supplementary Fig. 4** Comparable cytomembrane expression of CARs with intact or truncated CD3ζ endodomain. **a**, **b** CARs comprising a ζ chain endodomain that encompasses different ITAM(s) were designed, and T cells were engineered to co-translationally express a CAR and GFP. Cells were then subjected to FCM assays for the levels of GFP and cytomembrane-anchored CARs. Data are representative images and expressed as the means ± SD of three independent experiments. **P* < 0.05, ***P* < 0.01, ****P* < 0.001.


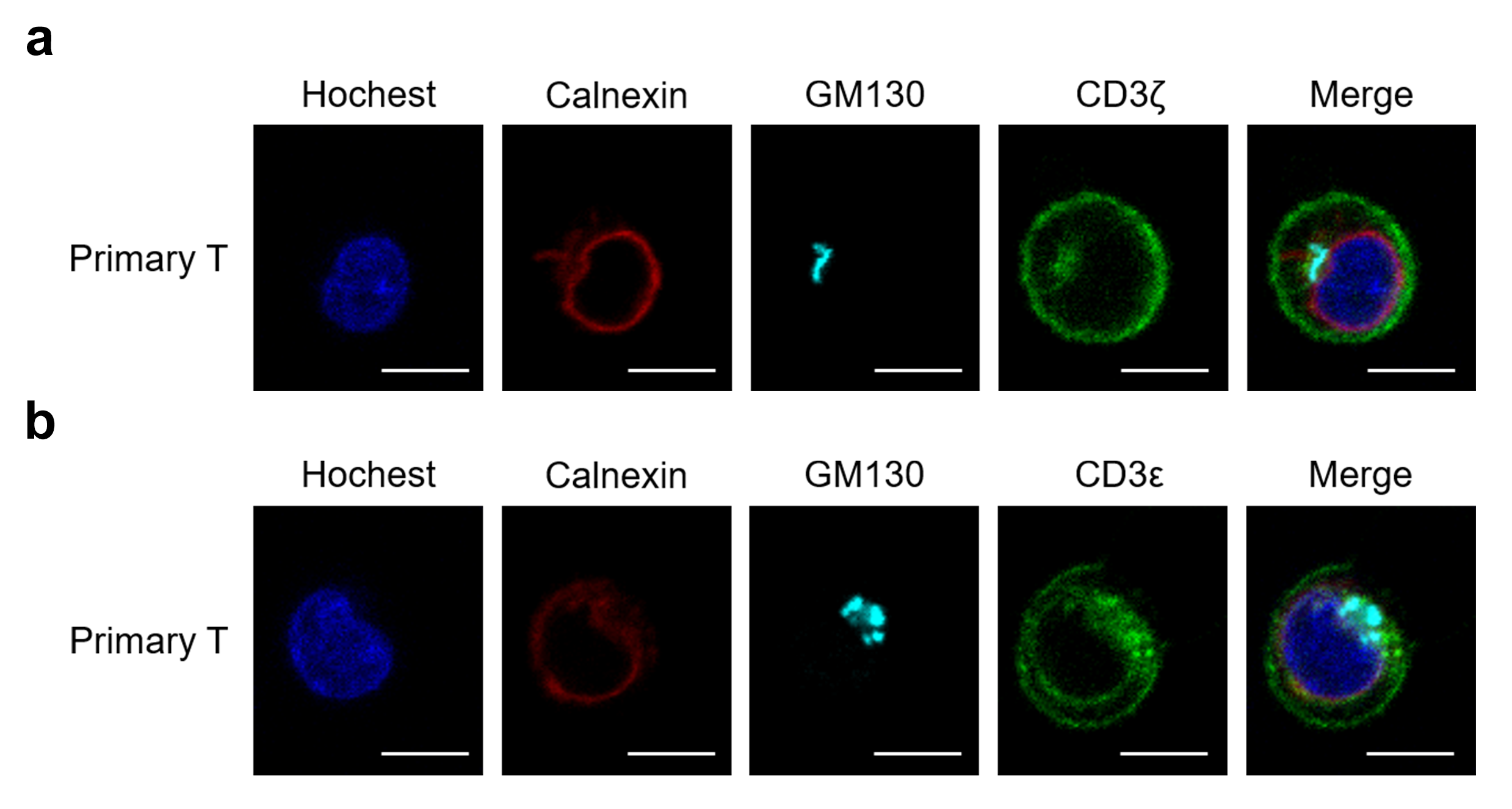


**Supplementary Fig. 5** Distinct localization of the ζ and ε subunits of CD3 in T cells. **a**, **b** Human T cells prepared from PBMC were subjected to immunofluorescence staining for indicated proteins. Calnexin and GM-130 are markers of the [endoplasmic reticulum](https://dict.cn/endoplasmic%20reticulum%20%28ER%29) and the Golgi apparatus, respectively. Bar, 10 μm. Data are representative images of three independent experiments.


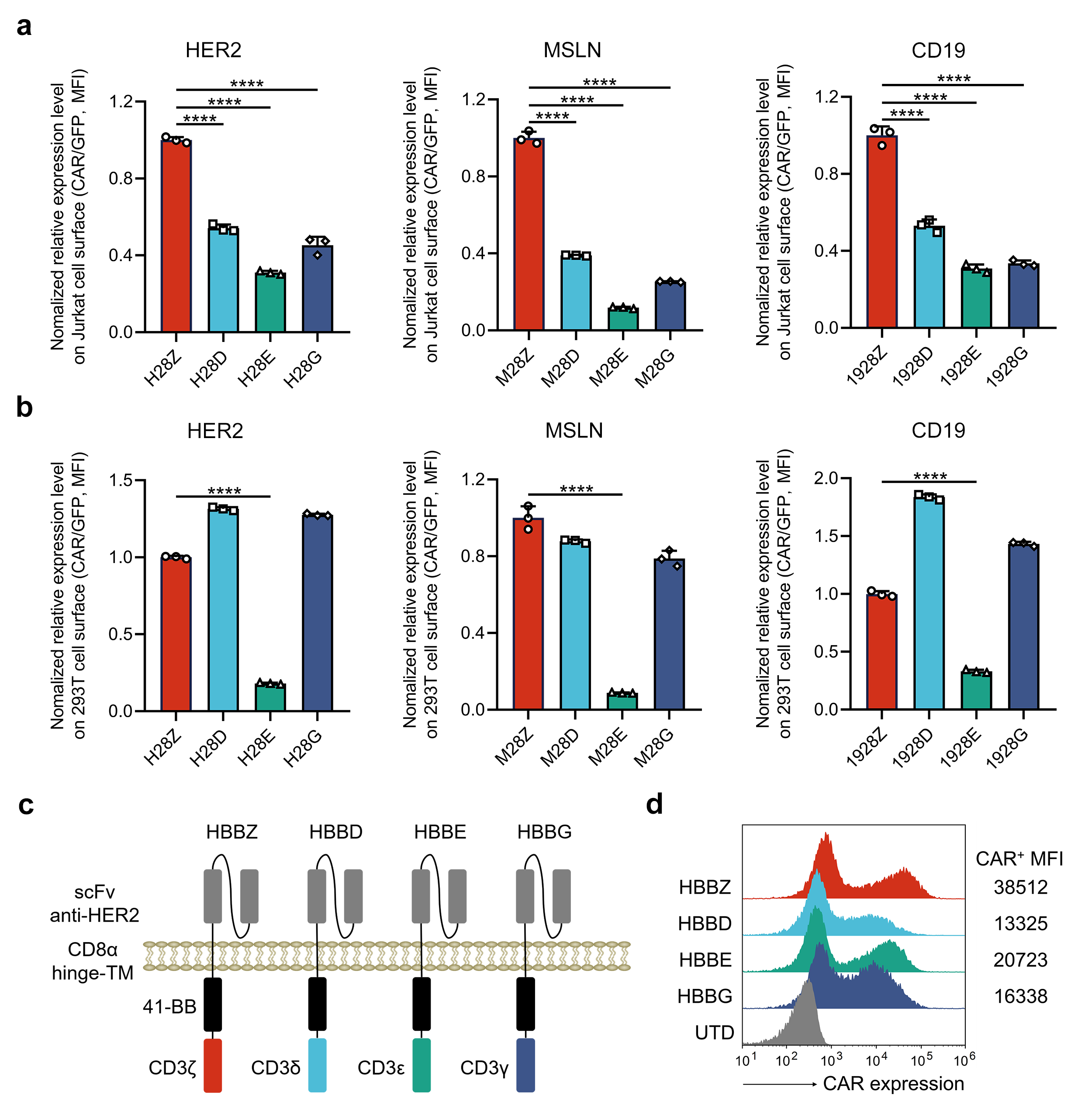


**Supplementary Fig. 6** Varied expression patterns of reconstituted CARs in Jurkat and HEK293T cells. **a**, **b** Constructs of CARs that target the indicated antigens were introduced into Jurkat (**a**) or HEK293T cells (**b**), and the cytomembrane levels of CARs were examined and normalized to levels of co-translated GFP. **c** Schematic diagram of CARs constructed using a HER2 scFv, a 4-1BB-derived costimulatory domain and varied endodomains. **d** Human T cells isolated from PBMC were infected with recombinant lentiviruses for indicated CARs, followed by FCM assay for cytomembrane CAR expression. Data are representative images and expressed as the means ± SD of three independent experiments. *****P* < 0.0001.

**
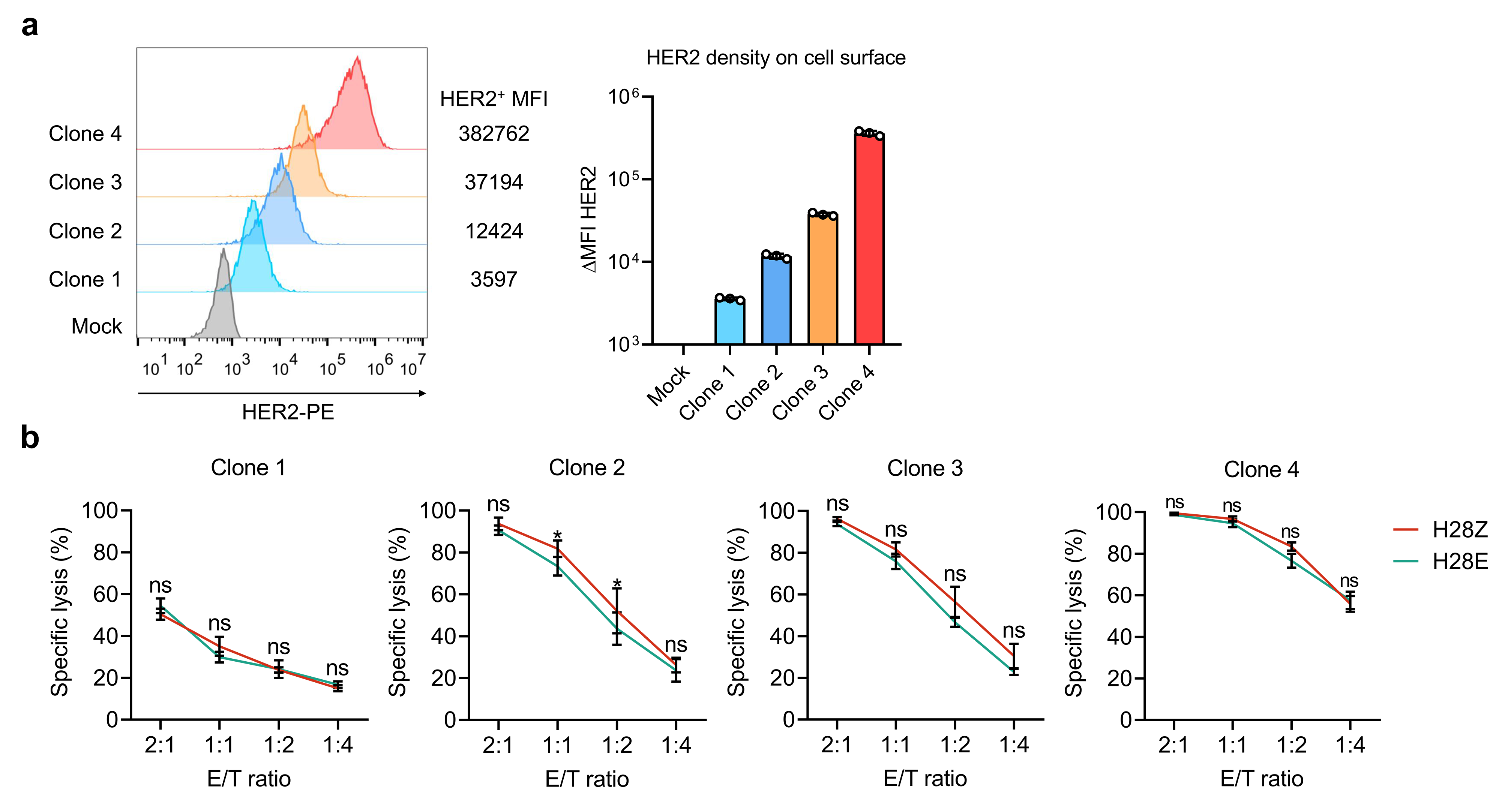
**

**Supplementary Fig. 7** H28E and H28Z CAR-T cells exhibited comparable cytolytic activities in a wide range of HER2 densities on neoplastic cells. **a**, **b** PC-9 cells were stably transfected to obtain cell clones expressing varied levels of HER2 as detected via FCM (**a**). These cell clones were then expanded and cocultured with indicated CAR-T cells for 16 h, and the percentages of cell lysis were calculated and plotted (**b**). **P* < 0.05; ns, non-significant.

**
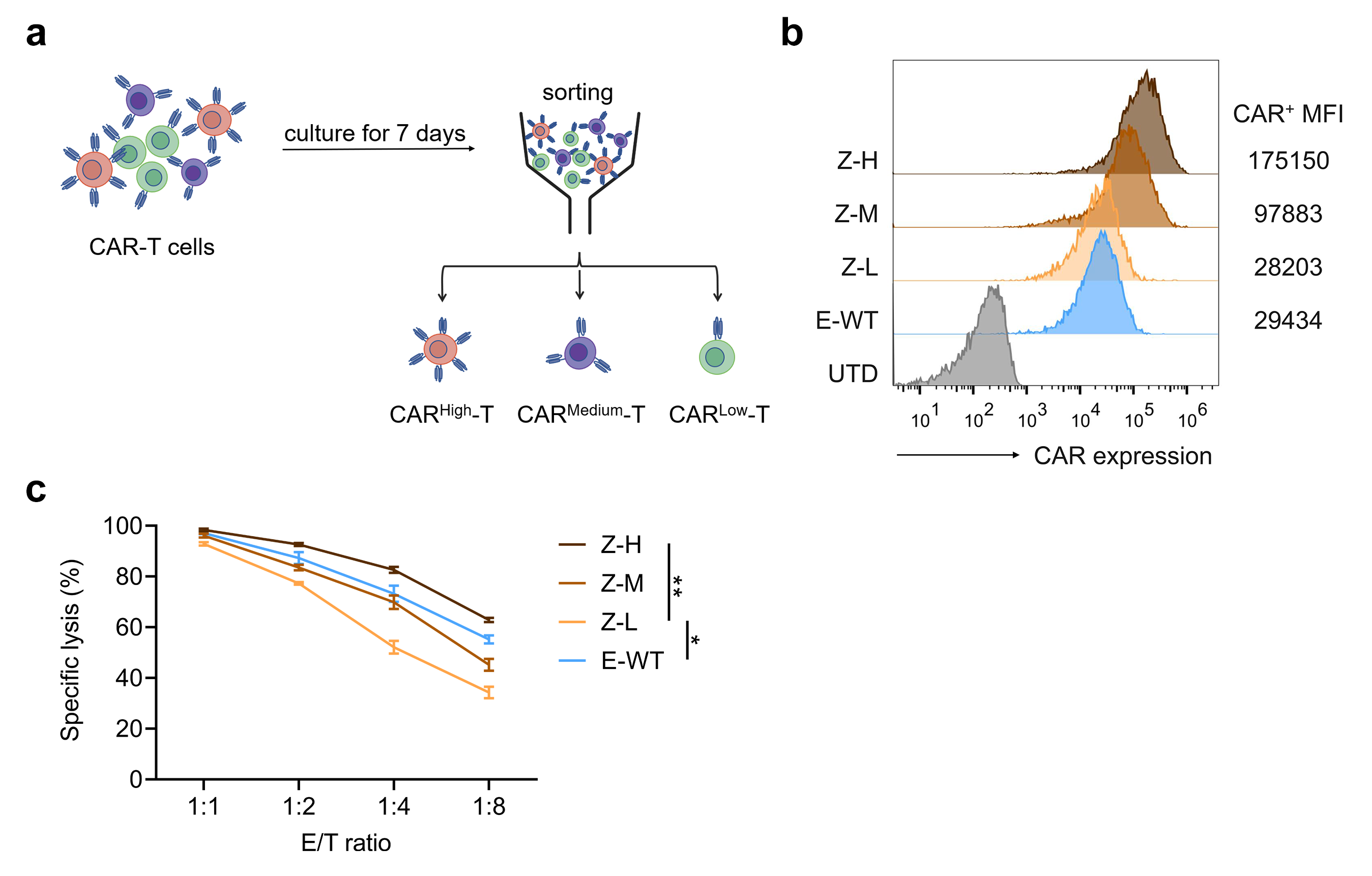
**

**Supplementary Fig. 8** CD3ε-based CAR confers T cells sufficient antigen-induced cytolytic activity despite modest cytomembrane expression. **a**-**c** Human T cells infected with recombinant lentiviruses expressing the H28Z CAR were further subjected to fluorescence-activated cell sorting (FACS) according to the levels of cytomembrane CAR (**a**, **b**). The sorted CAR-T cells or H28E CAR-T cells were cocultured for 16 h with PC-9 cells modified to co-express HER2 and luciferase, and cell lysis was evaluated via measurement of luciferase activities of the target cells (**c**). Z and E represents CD3 ζ chain- and ε chain-based CAR, respectively; H, high; M, medium; L, low. UTD, untransduced cells. Data are representative images and expressed as the means ± SD of three independent experiments. **P* < 0.05, ***P* < 0.01.

**
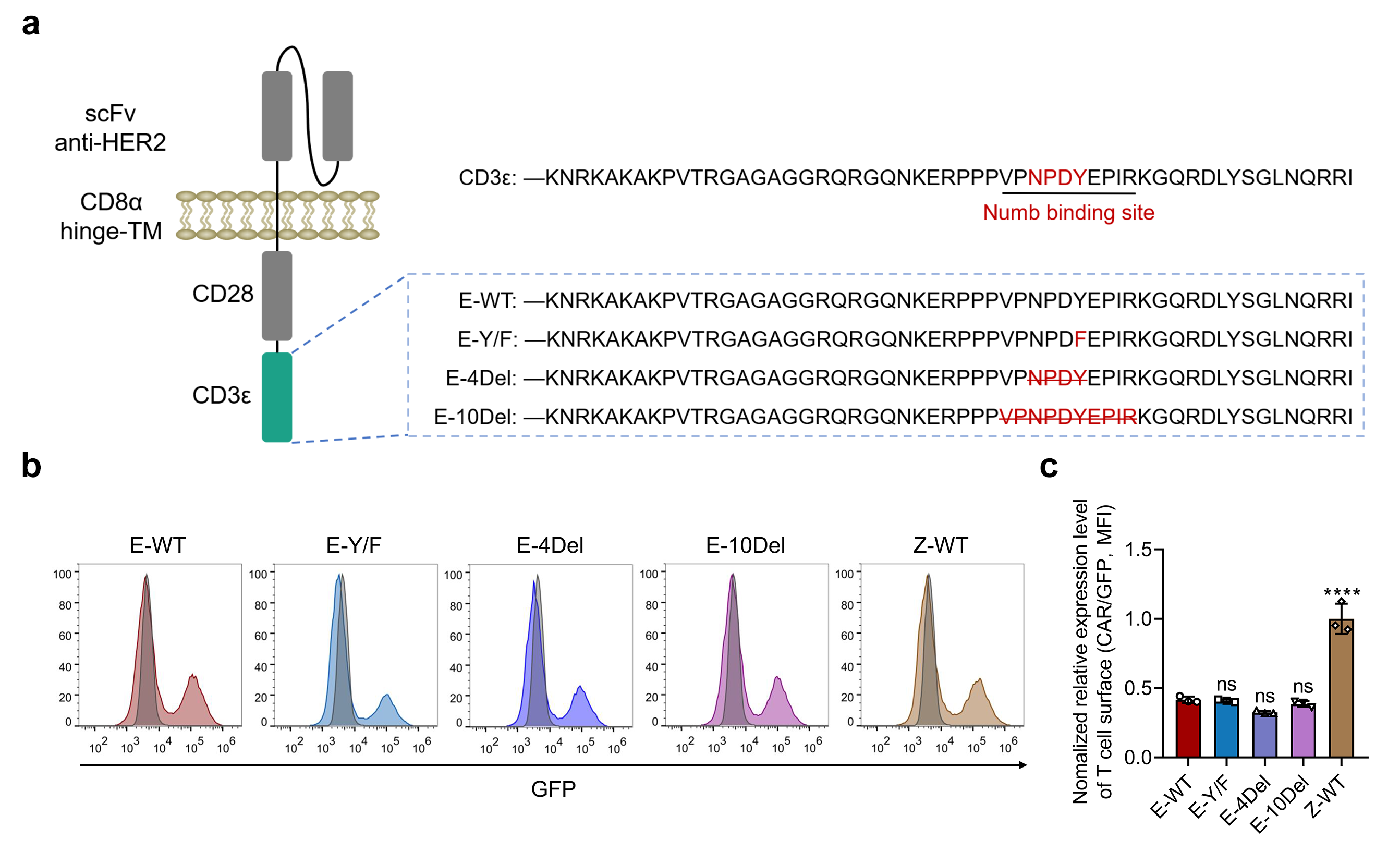
**

**Supplementary Fig. 9** Reduced surface display of CD3ε-based CAR was not attributed to Numb-mediated endocytosis. **a** Sequences for H28E CAR variants in which the reported Numb-binding sequences were mutated or deleted. **b**, **c** CARs described in (**a**) were introduced into T cells, and the surface levels of these CARs was assayed via FCM. Data are representative images and expressed as the means ± SD of three independent experiments. *****P* < 0.0001; ns, non-significant.

**
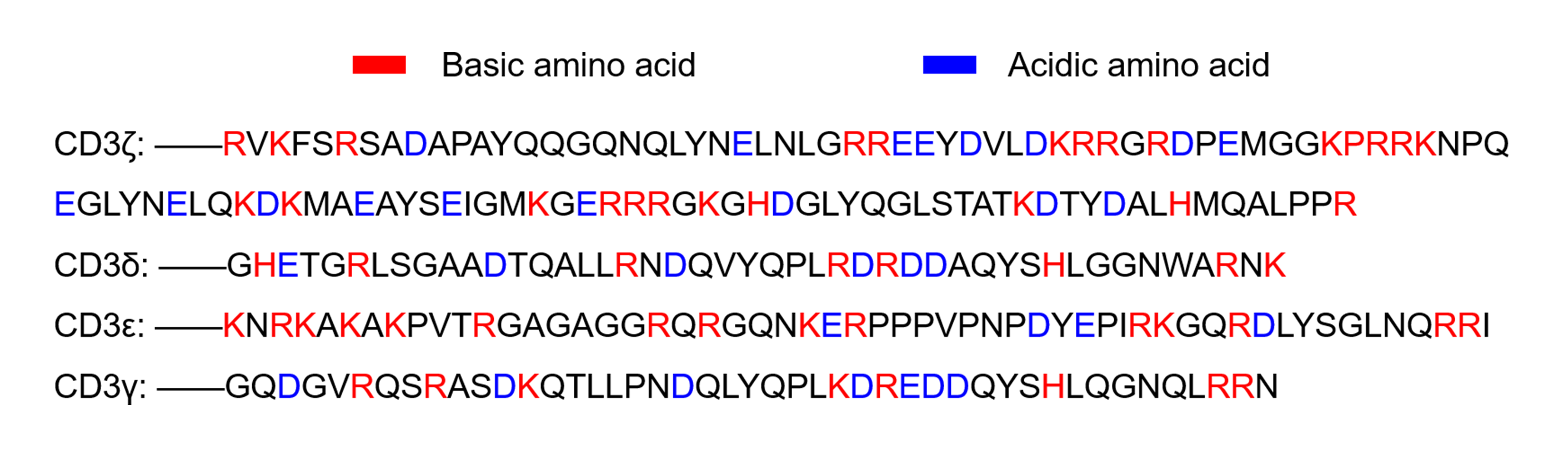
**

**Supplementary Fig. 10** Schematic diagram showing the basic (red) and acidic (blue) residues in the ICDs of CD3 subunits.

**
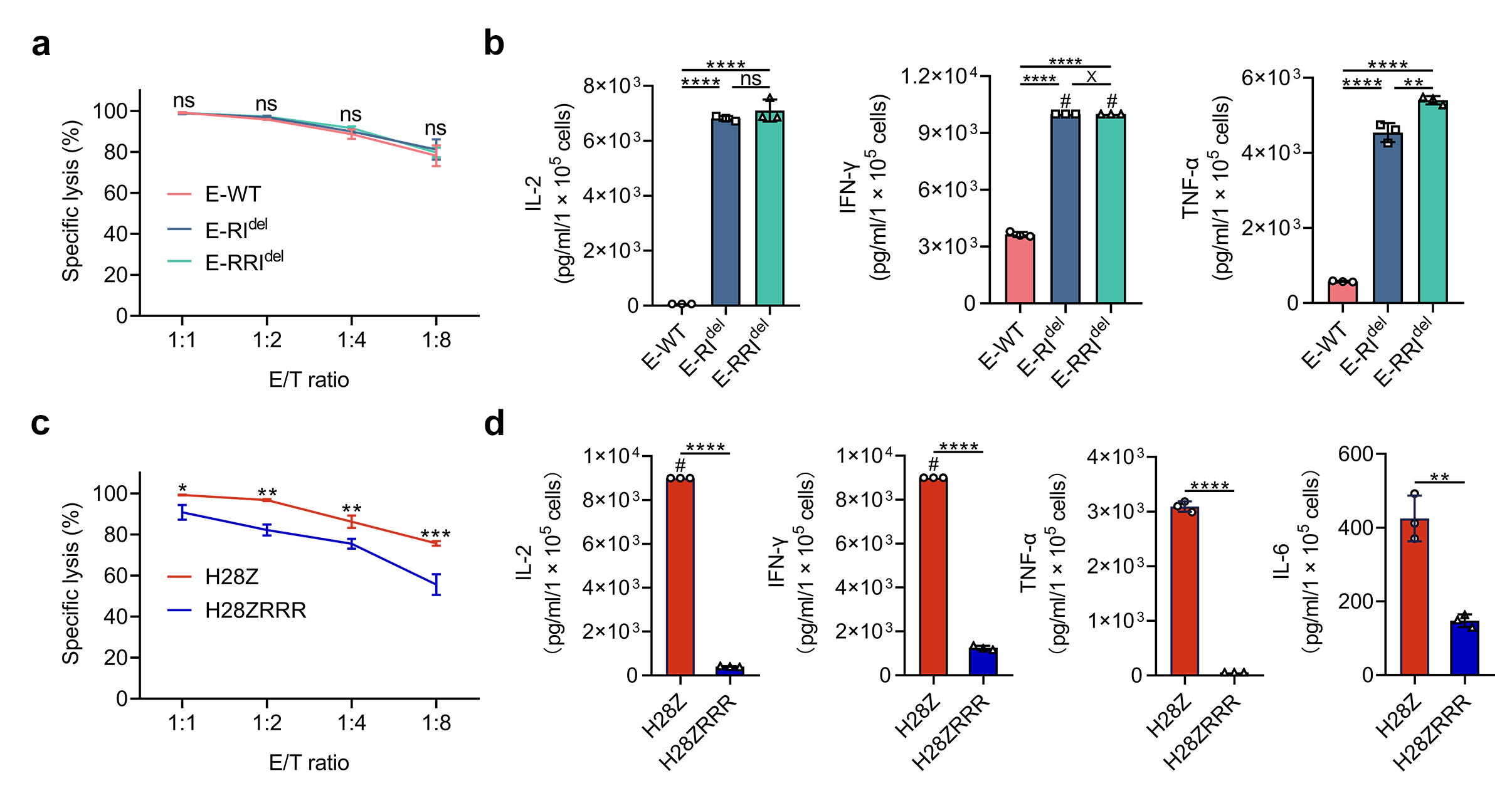
**

**Supplementary Fig. 11** Carboxyl-tail modification of CAR affects the characteristics of the derived CAR-T cells. **a**-**d** T cells expressing the modified CARs as illustrated in **Figure 5k** were generated. CAR-T cells were cocultured with SKBR3 cells for 16 h, and the percentages of cell lysis were calculated and plotted (**a**, **c**). For cytokine release assays, these CAR-T cells were cocultured with the HER2-overexpressing PC-9 cells (E:T = 1:1) for 24 h, and then subjected to FCM (**b**, **d**). **P* < 0.05, ***P* < 0.01, ****P* < 0.001, *****P* < 0.0001; #, concentrations beyond the measurable range; ×, uncomparable; ns, non-significant.


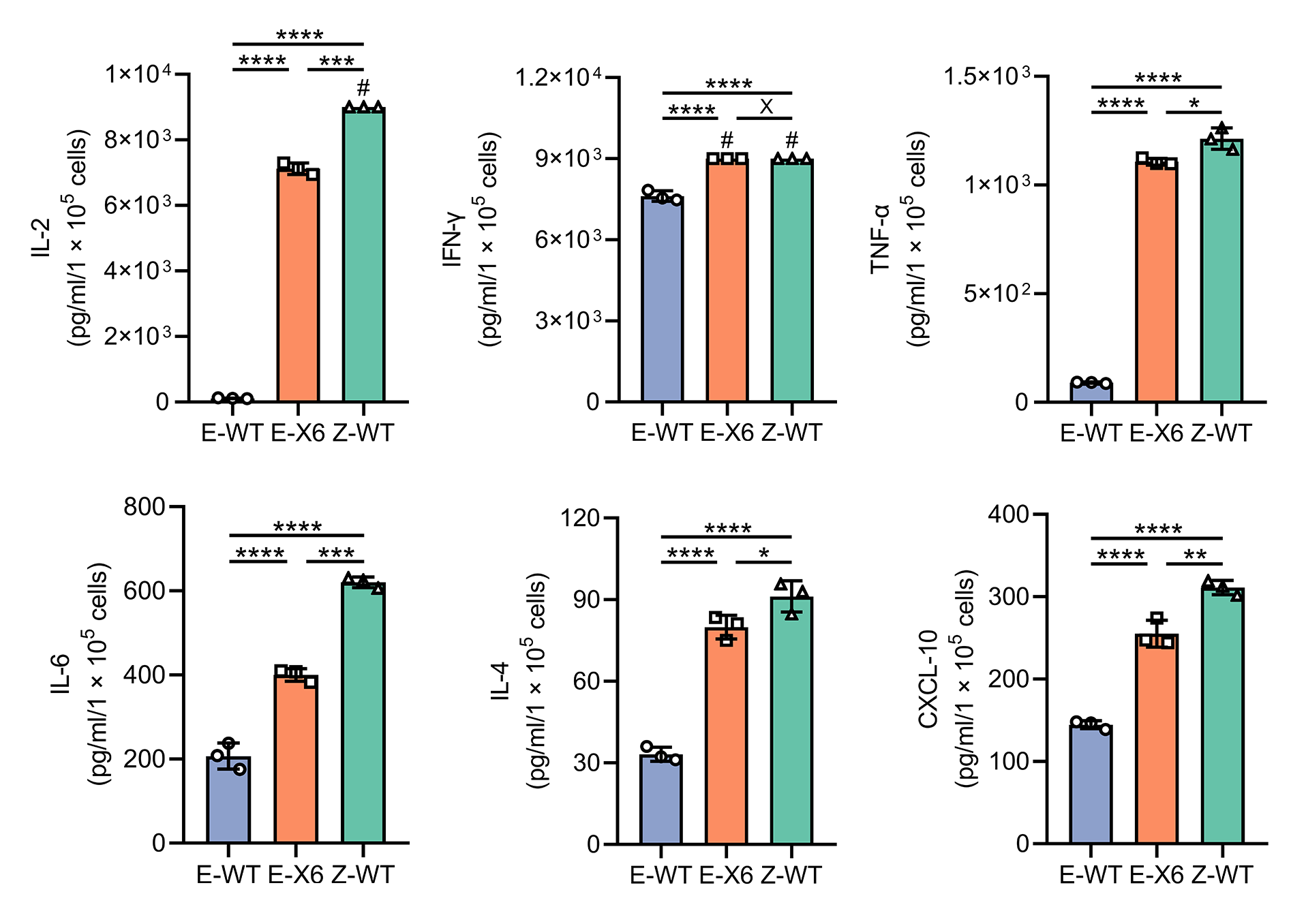


**Supplementary Fig. 12** Distinct cytokine production by T cells expressing different levels of ε or ζ chain-based CARs. T cells engineered with the indicated CARs were cocultured with HER2-overexpressing PC-9 cells (E:T = 1:1) for 24 h, and the levels of cytokines produced by CAR-T cells were measured and plotted. E-WT and Z-WT were CARs containing an intact endodomain of the CD3 ε and ζ chain, respectively, while E-X6 was a CAR comprising a truncated endodomain of CD3ε. Data are expressed as the means ± SD of three independent experiments. **P* < 0.05, ***P* < 0.01, ****P* < 0.001, *****P* < 0.0001; #, concentrations beyond the measurable range; ×, uncomparable.

**
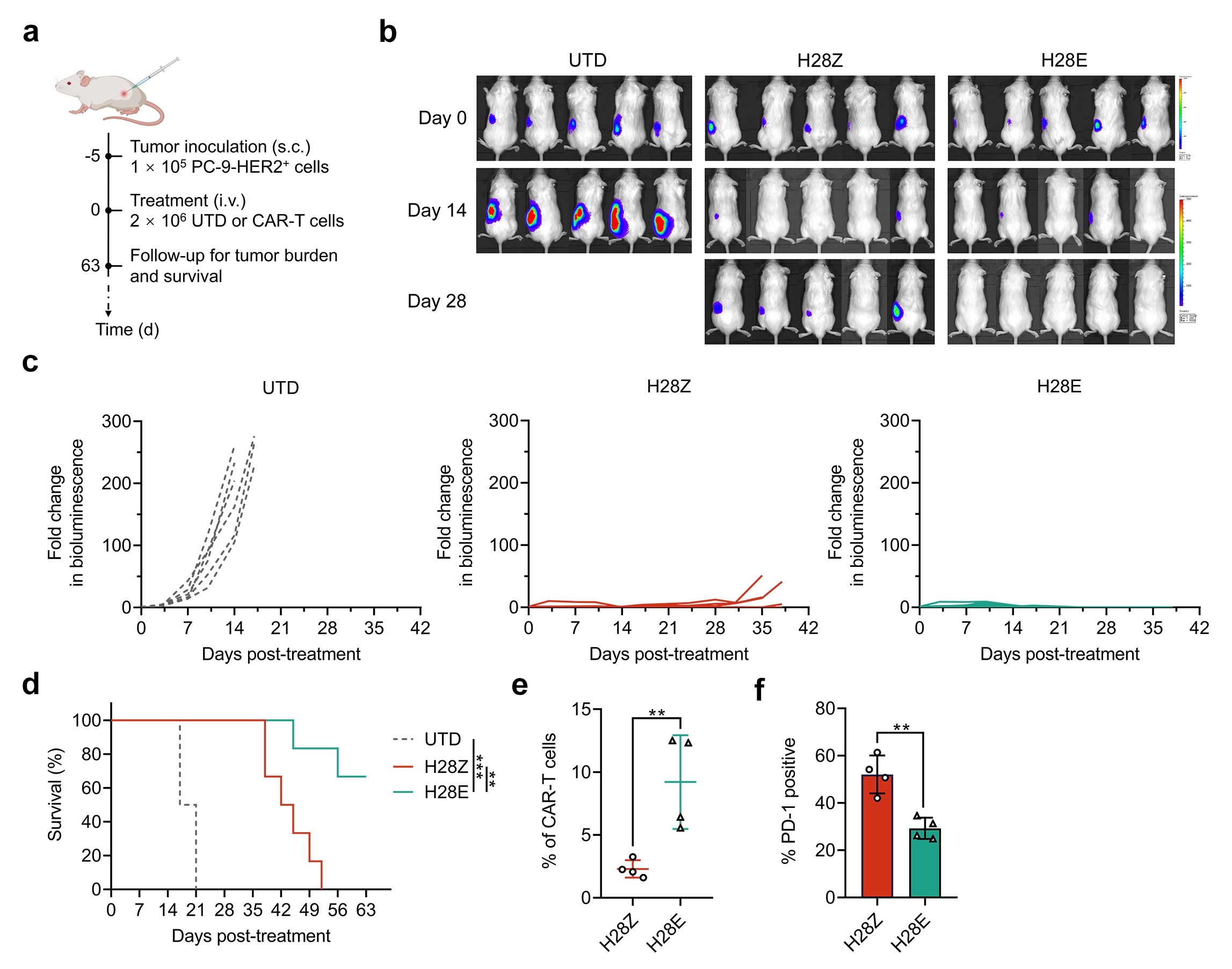
**

**Supplementary Fig. 13** Suppression of *in vivo* tumor growth by a single dose of CAR-T cells in a xenograft model. **a**-**f** Mice were inoculated with malignant cells and received injection with a single dose of CAR-T cells as described (**a**). Bioluminescent imaging for tumors was performed on indicated days after administration of CAR-T cells (**b**). Fold changes in bioluminescence were calculated (**c**; n = 6), and survival of mice receiving treatment with CAR-T cells was recorded and plotted (**d**; n = 6). The spleens of mice were separated, and splenocytes were subjected to FCM assays for a proportion of CAR-T cells (**e**; n = 4) and for a ratio of PD-1-positive population in CAR-T cells (**f**; n = 4). UTD, untransduced T cells. ***P* < 0.01, ****P* < 0.001.

**
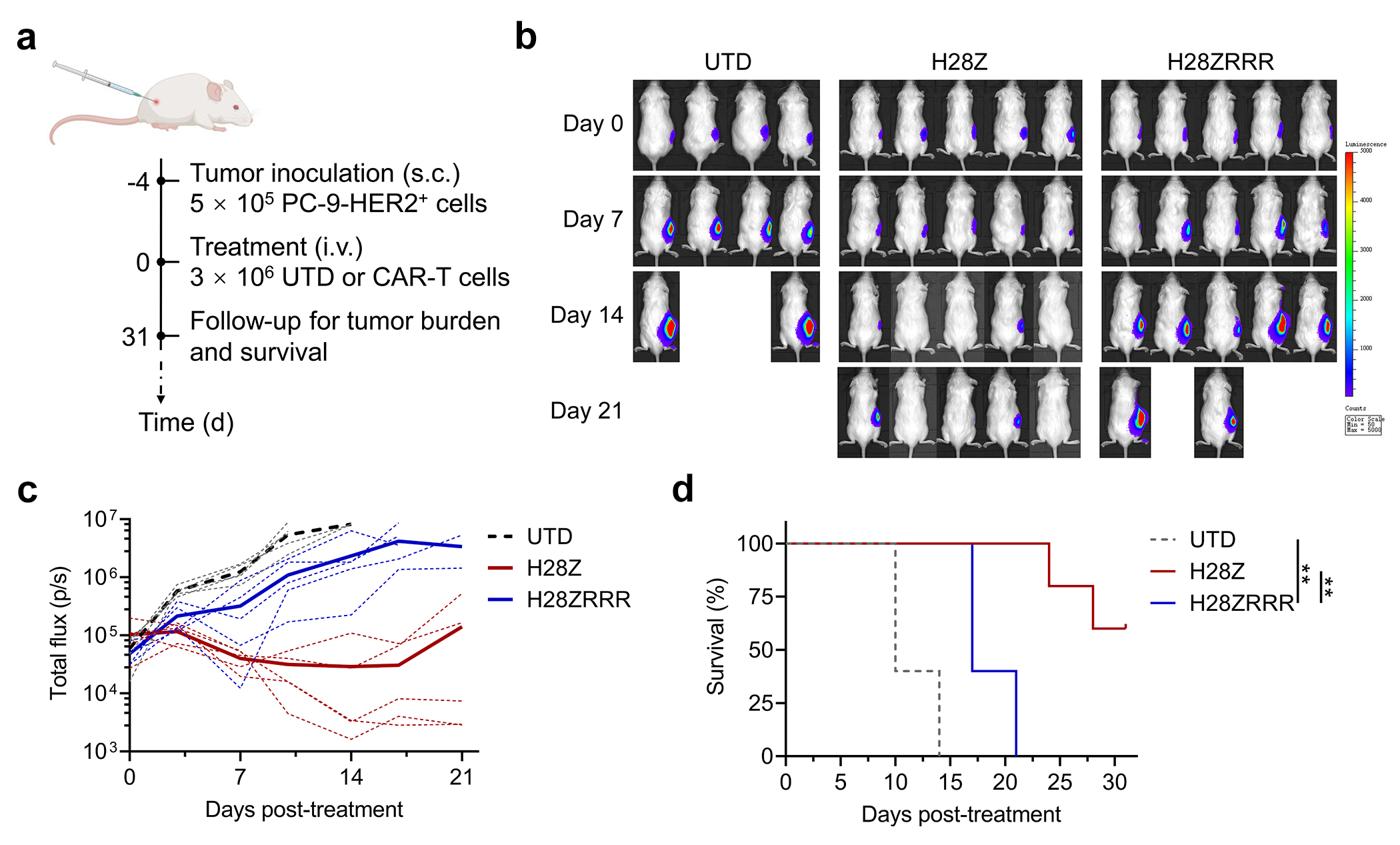
**

**Supplementary Fig. 14** Carboxyl tail modification of conventional CAR fails to improve the anti-tumor performance of CAR-T cells. **a**-**d** Human PC-9 cells transduced to overexpress HER2 were used for generation of a xenograft tumor model on B-NSG mice (**a**). Mice were then treated with the indicated CAR-T cells (n = 5). Bioluminescent imaging for tumors was performed on indicated days after administration of CAR-T cells (**b**), and fold changes in bioluminescence were calculated (n = 5) (**c**). The survival of mice receiving treatment with CAR-T cells was recorded and plotted (n = 5) (**d**). Data are expressed as the means ± SD of three independent experiments. ***P* < 0.01.


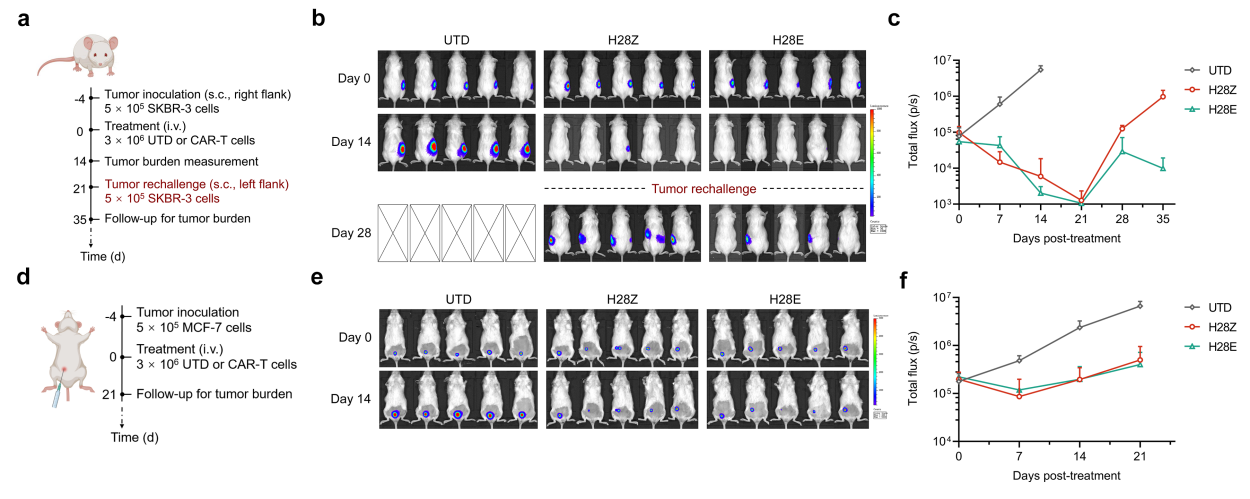


**Supplementary Fig. 15** Suppression of xenograft tumors expressing different levels of HER2 by CAR-T cells. **a**-**f** Human breast cancer SKBR-3 (**a**) and MCF-7 (**d**) cells that express varied levels of HER2 (shown in **Figure 4j**, **k**) were used for generation of xenograft tumor models on B-NSG mice. Mice were then treated with CAR-T cells. Where indicated, mice also received rechallenging with malignant cells for evaluation of a long-term tumor suppression. Bioluminescent imaging for tumors was performed on indicated days after administration of CAR-T cells (**b**, **e**), and fold changes in bioluminescence were calculated (**c**, **f**; n = 5).

**
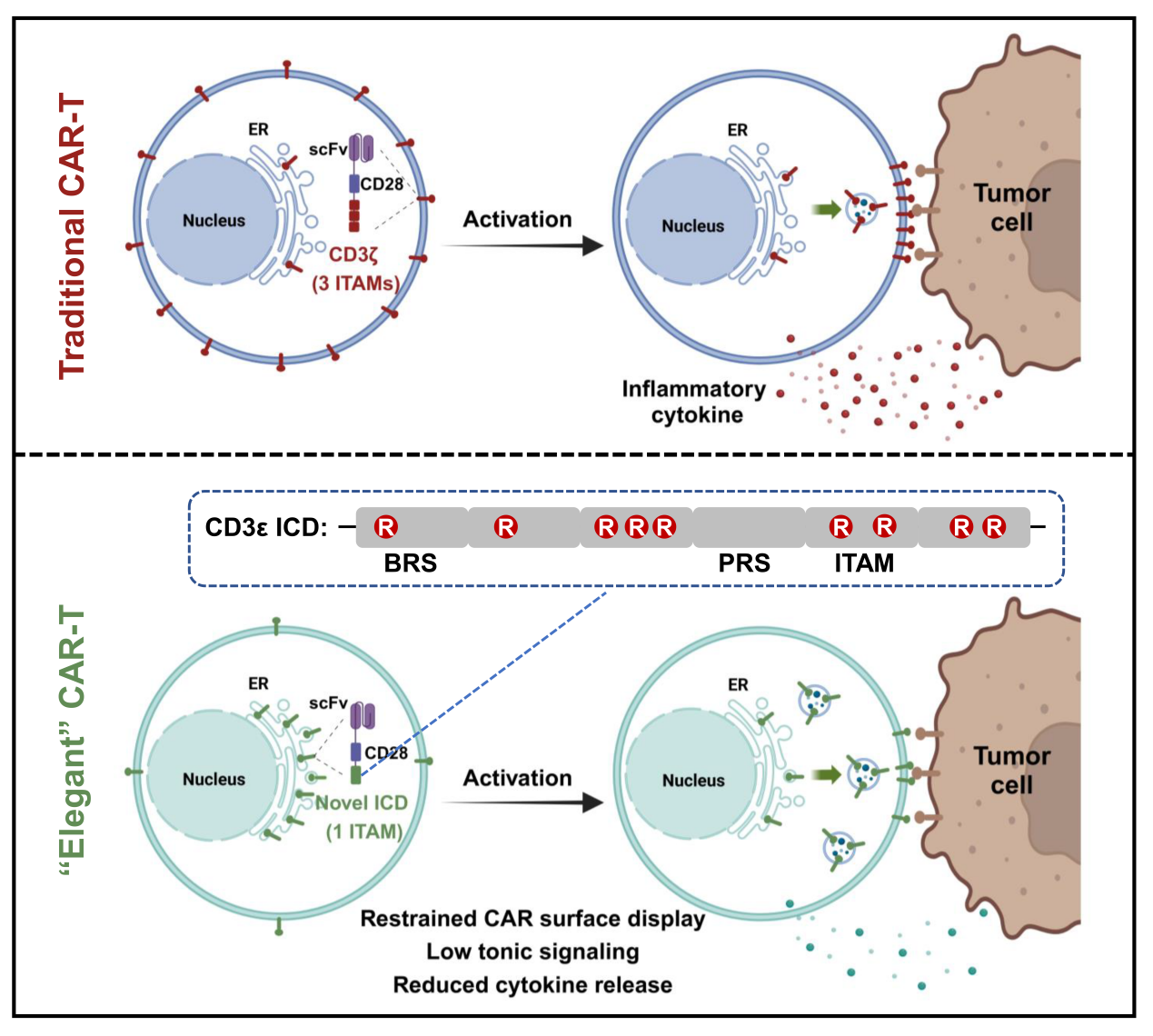
**

**Supplementary Fig. 16** A diagram depicting the superiority of CD3ε-based “Elegant” CARs to traditional CARs. Compared with traditional CD3ζ CARs, those composed of a CD3ε ICD harbor a single ITAM and trigger low detrimental tonic signal, enabling resistance of the engineered T cells to exhaustion. Meanwhile, the incorporated CD3ε domain mediates a limited cytomembrane expression of the CAR and contributes to reduced inflammatory cytokine release by CAR-T cells. These characters collaboratively warrant improved persistence of CAR-T cells against *in vivo* solid tumors. The illustration was generated via an online tool (BioRender.com).
